# Building blocks of joint attention: Early sensitivity to having one’s own gaze followed

**DOI:** 10.1016/j.dcn.2019.100631

**Published:** 2019-03-05

**Authors:** Holly Rayson, James J. Bonaiuto, Pier F. Ferrari, Bhismadev Chakrabarti, Lynne Murray

**Affiliations:** a School of Psychology and Clinical Language Sciences, University of Reading, Reading, United Kingdom; b Institut des Sciences Cognitives - Marc Jeannerod, CNRS, Bron, France; c Sobell Department for Motor Neuroscience and Movement Disorders, University College London, United Kingdom; d Department of Psychology, Stellenbosch University, South Africa; e Department of Psychology, University of Cape Town, South Africa

**Keywords:** Infant, Joint attention, EEG, Alpha, Preferential looking

## Abstract

Detecting when one’s own gaze has been followed is a critical component of joint attention, but little is known about its development. To address this issue, we used electroencephalography (EEG) to record infant neural responses at 6.5 and 9.5 months during observation of an adult either turning to look at the same object as the infant (congruent actor), or turning to look at a different object (incongruent actor). We also used a preferential looking paradigm to investigate whether infants would demonstrate a preference for the congruent versus incongruent actor. Greater suppression of alpha band activity in the congruent compared to incongruent condition was revealed at both ages in central and parietal regions. However, the effect of congruency on alpha suppression was stronger at 9.5 months, and only at this age did infants demonstrate a preference towards looking at the congruent actor. Together, these results suggest that although infants are sensitive to others’ gaze following from early on, important neural and behavioural developments occur between 6.5 and 9.5 months.

## 1 Introduction

Essential for everyday social interactions,’ joint attention’ (JA) involves the triadic coordination of attention between self, other, and environment. JA-relevant behaviours emerge between 3 and 18 months (Bakeman and Adamson, 1984; Butterworth, 2001; Carpenter et al., 1998; D’Entremont et al., 1997; Mundy et al., 2007), although the age at which these are driven by an awareness of others’ visual perspective or intentionality is debated (Corkum and Moore, 1998; Tomasello et al., 2005). The ability to engage in JA represents a critical milestone in early development, and has been linked to the subsequent acquisition of many complex socio-cognitive skills. These include cooperative behaviour, theory of mind (ToM), and language learning (Baron-Cohen, 1991; Brooks and Meltzoff, 2015; Morales et al., 2005; Mundy et al., 2007; Tomasello et al., 2005), with JA impairment also one of the earliest indicators of autism spectrum disorder (ASD) (Charman, 2003; Charman et al., 2000).

JA can be divided into two main subtypes: ‘responding to joint attention’ (RJA), which involves following another individual’s gaze and/or gestures; and ‘initiating joint attention’ (IJA), which comprises the use of one’s own gaze and/or gestures to direct someone else’s attention. Both result in the sharing of a common point of reference (Billeci et al., 2016; Eggebrecht et al., 2017; Mundy, 2018; Mundy and Newell, 2007; Redcay et al., 2012; Seibert et al., 1982). RJA and IJA reflect partially dissociated processes, differing somewhat in their developmental trajectory (Beuker et al., 2013; Mundy et al., 2007), and making independent contributions to the emergence of specific abilities in later childhood (Mundy and Jarrold, 2010). In the adult brain, JA recruits widespread cortical and subcortical networks, including attentional, social perception, and visual circuitries (Caruana et al., 2015; Oberwelland et al., 2016; Redcay et al., 2012). Both overlapping and distinct regions are active during RJA and IJA (Mundy and Jarrold, 2010; Redcay et al., 2012; Schilbach et al., 2010), with reward-related areas linked specifically to the latter (Gordon et al., 2013; Schilbach et al., 2010). In older children and adolescents, both processes recruit brain regions similar to those activated in adults (Oberwelland et al., 2016), but whether this is the case in younger individuals is less clear, especially in the case of IJA. One recent study has associated IJA with a fairly distributed system in 12–24 month olds, comprising default mode, dorsal attention, and somatomotor networks (Eggebrecht et al., 2017). At similar ages, resting-state electroencephalography (EEG) power has been linked to IJA in anterior brain regions, but to RJA in more posterior regions (Mundy et al., 2000). This is in keeping with the hypothesized involvement of anterior and posterior attention networks in IJA and RJA, respectively (Mundy and Newell, 2007).

Crucially, many questions remain concerning the precursors of JA in the very first months of life. Since IJA may be a more sensitive index of socio-cognitive development than RJA (Tomasello, 1995), as well as a more robust symptom of neurodevelopmental disorders such as ASD (Mundy, 2018; Mundy et al., 2016), research aimed at elucidating the early building blocks of IJA is of particular importance. Accordingly, our study was designed to investigate the emergence of a critical, but largely neglected, component of IJA: detecting when one’s own gaze has been followed (Stephenson et al., 2018). Essential for determining whether or not one has been successful in directing someone else’s gaze, an early sensitivity to having one’s gaze followed is likely foundational for the *intentional* directing of another’s attention later on in infancy (Mundy, 2018). In other words, an early ability to detect implicitly the congruency between own gaze behaviour and that of a social partner represents an important building block for the development of ‘true’ IJA, which is characterized by intentionality and an awareness of others’ mental states. Near-infrared spectroscopy (NIRS) research suggests that left frontal brain regions exhibit such sensitivity from around half-way through infants’ first year (Grossmann et al., 2013), but to our knowledge, no other study has explored this to date. Nothing is known about how the capacity to detect others’ gaze following may develop in the months leading up to overt IJA-related behaviour (e.g. pointing and adult-object gaze alternation around 10–12 months; Mundy et al., 2007; Tomasello, 1995), how such sensitivity may be reflected over wider cortical areas, or how this may relate to behavioural responses.

We used EEG to record infant neural activity during observation of an adult actor following their gaze at both 6.5 and 9.5 months of age. In adults, event related desynchronization (ERD) occurs in the alpha frequency band after leading and following someone else’s gaze in central, parietal, and occipital regions (Lachat et al., 2012). In infants, widespread alpha ERD has also been observed during concurrent gaze to an object after an adult has made eye contact with them (Hoehl et al., 2014). Additionally, attenuation in the alpha band is associated with a number of JA-relevant processes such as interpersonal synchronization (Dumas et al., 2010; Novembre et al., 2016), sustained attention (Xie et al., 2018), joint action (Meyer et al., 2011), action recognition (Ulloa and Pineda, 2007), and ToM (Pineda and Hecht, 2009), therefore we focused our analyses on activity in this band.

We also included a preferential looking paradigm in our experiment to explore whether infants would show a bias towards looking at an actor who had previously followed their gaze. In adulthood, having one’s own gaze followed affects how a social partner is perceived and how that partner is responded to (Bayliss et al., 2013; Grynszpan et al., 2017; Willemse et al., 2018). Similar to the effects of imitation (Chartrand and Bargh, 1999; Hove and Risen, 2009; Neufeld and Chakrabarti, 2016; Stel and Vonk, 2010), adults favour others who follow their gaze and rate them as more pleasant (Bayliss et al., 2013; Grynszpan et al., 2017; Willemse et al., 2018). JA shares many characteristics of imitation (Hoffman et al., 2006; Lachat et al., 2012; Triesch et al., 2007), and the rewarding experience of having a social partner gaze in the same direction as oneself has been directly compared to that of being imitated (Edwards et al., 2015). In support, both social imitation and having one’s gaze followed recruit reward-related brain regions in adults (Gordon et al., 2013; Hsu et al., 2017; Schilbach et al., 2010). The use of gaze biases as a proxy for relative reward value has been well demonstrated, with both adults and infants preferring to look at someone who imitates them versus someone who does not (Agnetta and Rochat, 2004; Meltzoff, 1996, 1990; Neufeld and Chakrabarti, 2016). Whether individuals show a similar preference for looking towards someone who follows their gaze, however, is unknown.

Based on the above, we tested four main hypotheses concerning an early sensitivity to having one’s own gaze followed: i) that differences in infant alpha power would be found in central, parietal, and left-frontal electrode clusters during observation of an adult following their gaze (congruent condition) versus another adult looking in the opposite direction (incongruent condition) (Grossmann et al., 2013; Hoehl et al., 2014; Lachat et al., 2012); ii) that these differences in alpha power between the congruent and incongruent conditions would be more pronounced at 9.5 compared to 6.5 months (Mundy et al., 2007; Tomasello, 1995); iii) that no differences in alpha power between conditions would be found in occipital electrode clusters (visual alpha) due to the similarity between conditions in terms of low-level visual features (Rayson et al., 2017, 2016); and iv) that infants would demonstrate a preference towards looking at the adult who had previously followed their gaze versus the one who had not (Agnetta and Rochat, 2004; Meltzoff, 1996, 1990; Neufeld and Chakrabarti, 2016).

## 2 Materials and methods

### 2.1 Participants

A total of 23 infants (13 male, 10 female) aged 6.5 months (M = 200.91 days, SD = 5.86) and 24 infants (11 male, 13 female) aged 9.5 months (M = 292.92 days, SD = 7.88) were included in the final sample for analysis. More details concerning participants and exclusions prior to analysis can be found in the Supplementary Information (SI). The study was approved by the University of Reading Research Ethics Committee (31.07.14), with participants recruited from the ‘Child Development Database’ maintained by researchers in the University’s School of Psychology and Clinical Language Sciences. Infants’ mothers gave written, informed consent before participation, and all research was conducted in accordance with the Declaration of Helsinki.

### 2.2 Preferential looking and gaze following stimuli

**Fig. 1:**
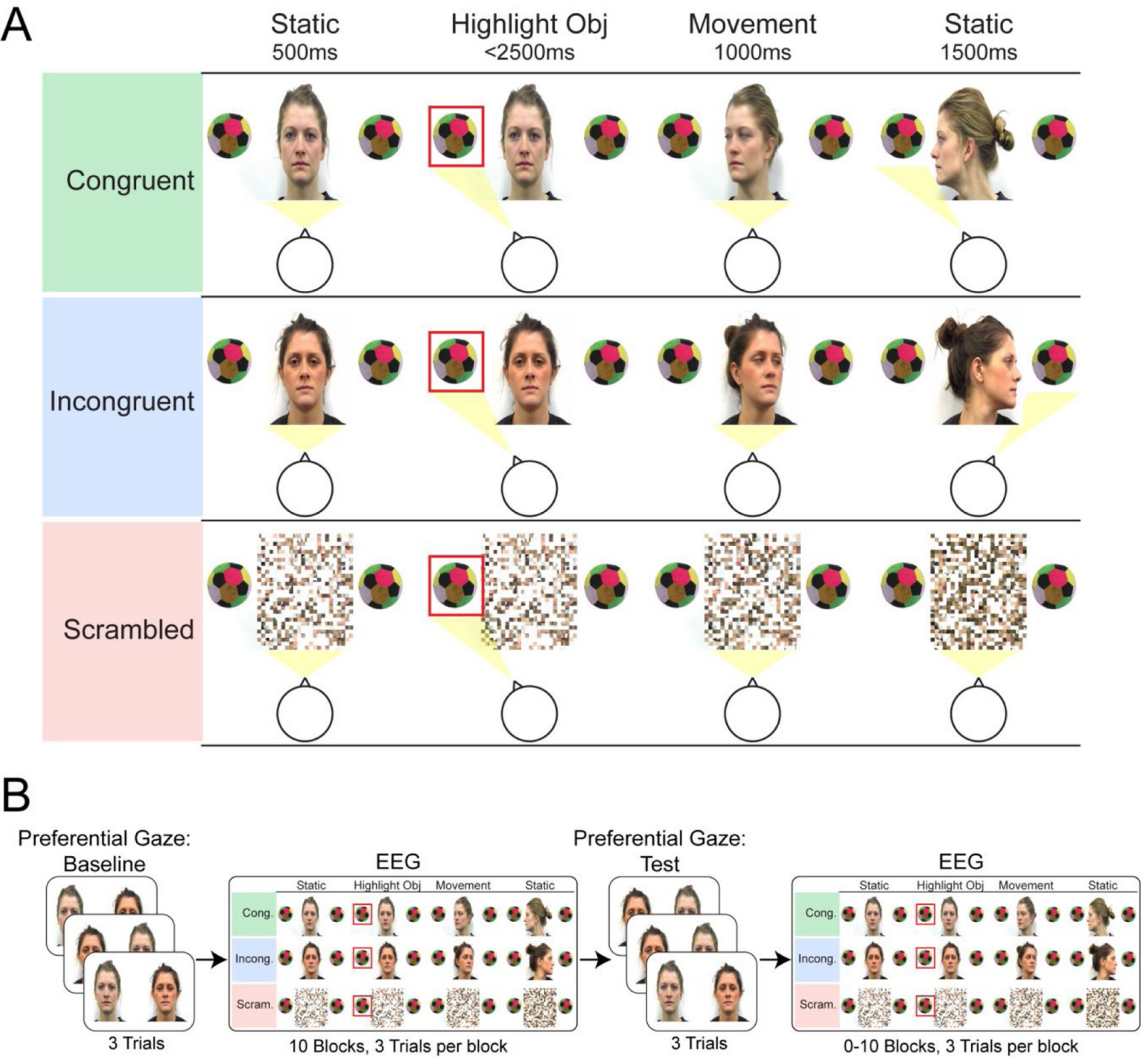
*A) Time-course of the gaze following stimuli in each condition (congruent, incongruent, and scrambled).* Each condition began with a static adult face (or scrambled face) with two identical objects on either side. After 500 ms, either the left or the right object was cued by a flashing red square, and once infants directed their gaze to the cued object, the adult actor either turned their head toward the cued or uncued object (i.e. adult target). The movement lasted 1000 ms and was followed by a 1500 ms static period. Typical gaze behaviour for the infant participant is shown below the stimuli for each condition: in the congruent and incongruent conditions, infants tended to look from the cued (highlighted) object to the adult, then to the adult’s target object; in the scrambled condition, infants tended to look from the cued (highlighted) object to the scrambled face only. *B) Order of preferential and gaze following (EEG) stimuli.* The whole experiment consisted of the following: a baseline block of preferential gaze trials (3 trials), followed by 10 blocks of gaze following trials (3 trials per block), a preferential gaze test block (3 trials), and then ≤ 10 blocks of gaze following trials (3 trials per block). EEG data were recorded during all gaze following trials (For interpretation of the references to colour in this figure legend, the reader is referred to the web version of this article).

Preferential looking stimuli consisted of static images of two adult actors (both female) presented side-by-side (see Fig. 1: B). Each stimulus was displayed for 5000 ms per trial, eyes facing towards the infant. Video recordings were made of infants throughout the experiment, and from these, infant gaze towards the two actors during preferential looking trials was manually coded.

Gaze following stimuli consisted of short video clips featuring the two actors from the preferential looking stimuli. All clips began with 500 ms of a static face presented in the centre of the screen. Located on either side of this face were two identical objects (colourful balls), which were displayed throughout the trial. After the static period, one of the two objects was highlighted by a flashing red square, which was jittered up and down slightly in order to attract the infant’s attention. This attention grabbing sequence lasted for a maximum of 2500 ms, and if the infant looked at the highlighted object within this time, one of three experimental conditions followed: congruent, incongruent, or a scrambled control (see Fig. 1: A for the time-course of these stimuli). In the congruent condition, the actor turned to look at the object the infant had just looked towards (the previously highlighted ‘cued object’), but in the incongruent condition, the actor turned to look at the object on the opposite side to where the infant had just looked (the ‘uncued object’). The exogenous cuing of only one object at the start of each video allowed us to: a) balance the number of trials in which infants looked to the left or right object (overall, with each actor, and in each condition); and b) ensure that the infant focused on one object rather than quickly alternating their gaze between the two before the adult head turn began. At the start of the congruent and incongruent videos, actors were looking directly at the infant. For each participant, one actor always turned in the congruent direction, whereas the other actor always turned in the incongruent direction. The identity of the congruent/incongruent actor was counterbalanced across infants. Scrambled versions of the congruent and incongruent videos made up the control condition (i.e. a scrambled version of each left/right and actor1/actor2 versions of the congruent and incongruent videos). We chose to use the scrambled stimuli in order to control for overall motion across all experimental conditions, and explore any specificity of infant responses to head turns versus coherent motion in general. More information concerning scrambled stimuli can be found in the SI, and example stimuli are available as supplementary material.

In all gaze following conditions, the head turn/scrambled movement lasted for 1000 ms, with the end position held for a further 1500 ms (Fig. 1: A). Before each trial, a colourful moving pattern was displayed in the middle of the screen for 1000 ms. The video recordings of infants during the experiment were utilized to code infant gaze to various areas of interest (AOIs) during the gaze following trials; cued object/uncued object/adult face/adult target object (i.e. the object the adult turned to look at).

### 2.3 Design and procedure

During the experiment, infants were seated on mothers’ laps approximately 65 cm from a computer monitor. Stimuli were presented on the monitor using PsychoPy v1.80.04 (Peirce, 2008). At the start of the experiment, infants were presented with one block of preferential looking stimuli (three trials; baseline block), with the position (left/right) of the congruent and incongruent actors randomized across participants. This was followed by the gaze following stimuli, which were presented in blocks of three video clips (Fig. 1: B; one congruent, one incongruent, one scrambled). Presentation of these clips was randomized within blocks, and block order was randomized between participants. After 10 blocks of gaze following trials, infants were presented with another block of preferential looking trials (three trials; test block), followed by ≤ 10 blocks of gaze following trials (Fig. 1: B). Experimental blocks began when triggered manually by an experimenter, who was watching the infant live on a screen in another section of the room. Adult head-turns in the gaze following stimuli were also triggered by the experimenter, and only if infants looked towards the highlighted object within the 2500 ms attention-grabbing time-window. Information concerning how often infants looked to the cued object can be found in the SI. The inter-stimulus interval was randomized between 800 and 1200 ms. The experiment was terminated if infants became very inattentive, distressed, or started moving excessively.

### 2.4 EEG acquisition and analysis

EEG was recorded using a 128-channel Hydrocel Geodesic Sensor Net (EGI, Corp., Eugene, OR). Data were sampled at 250 Hz with an analogue band-pass filter of 0.1–100 Hz, and were recorded with the vertex as a common reference. Impedances were kept below 50 kΩ. Synchronous video recordings of the experiment (30 frames per second) were examined offline to allow exclusion of EEG trials in which the infant was inattentive or moving, and to facilitate the coding of infant gaze during both gaze following and preferential looking trials. See SI for details regarding pre-processing of the EEG data.

To compare power relative to baseline in the alpha frequency band, we analysed total-induced event related activity for each condition. Time-frequency decompositions were computed for each trial using built-in EEGLAB procedures with a fast Fourier transform using a 1-second Hann window with 50% overlap in 1 Hz bins from 2 to 35 Hz. We then averaged over trials within each condition, and then across the frequency bins of interest.

**Fig. 2:**
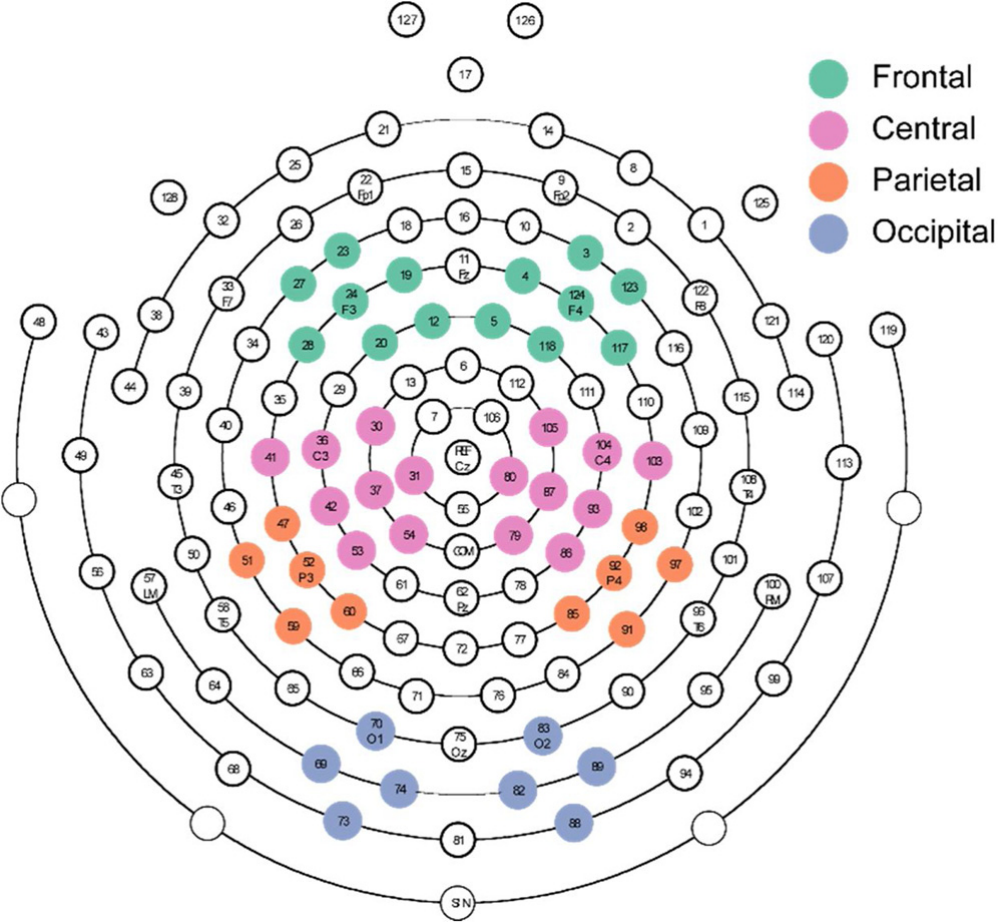
Clusters of electrodes used in the analysis of alpha power.

In each condition (congruent/incongruent/scrambled), changes in power from baseline were computed in the alpha frequency band. This was calculated as a relative change from baseline expressed as a percentage (X-B)/B*100, where X is alpha power averaged over the time window of interest and B is alpha power averaged over the baseline time period (Pfurtscheller and Aranibar, 1979). Negative baseline-corrected alpha power therefore indicates alpha event-related desynchronization (ERD), while positive values indicate alpha event-related synchronization (ERS). A 5–8 Hz band was used for 6.5-month-olds and 6–9 Hz for 9.5-month-olds, corresponding to the typical ranges used with these age groups and the increasing alpha peaks previously identified over these months (Cannon et al., 2016; Marshall et al., 2002; Michel et al., 2015; Nyström, 2008; Nyström et al., 2011). Changes in alpha power were computed over six windows of interest (WOIs): 0–500 ms, 500–1000 ms, 1000–1500 ms, 1500–2000 ms, 2000–2500 ms, and 2500–3000 ms after the onset of the observed adult head turn, allowing us to look at the timing of alpha activity changes. This was normalized as the percent change from the condition-specific (averaged across trials in that condition) baseline averaged over 100-400 ms of the static period at the start of a trial (Pfurtscheller and Aranibar, 1979). Based on other EEG studies of alpha band activity (Cannon et al., 2016; de Klerk et al., 2015; Saby et al., 2012; Umiltà et al., 2012), changes in alpha power were calculated for eight clusters of electrodes: two frontal clusters (left/F3, right/F4); two central clusters (left/C3, right/C4); two parietal clusters (left/P3, right/P4); and two occipital (left/O1, right/O2) (see Fig. 2). For each cluster, in each experimental condition and WOI, baseline-corrected alpha values were calculated for each subject. Statistical outlier segments were calculated and removed for each participant using methods established in other infant EEG research (Cannon et al., 2016; Saby et al., 2012); i.e. values greater than 1.5 times the interquartile range from the median were considered outliers.

A linear mixed model framework was used for statistical analysis using R (v3.4.2; R Development Core Team, 2011) and the lme4 package (Bates et al., 2014). Baseline-corrected alpha power was treated as the dependent measure, with condition (congruent/incongruent/scrambled), cluster (F3 / F4 / C3 / C4 / P3 / P4 / O1 / O2), WOI (0–500 / 500–1000 / 1000–1500 ms / 1500–2000 ms / 2000–2500 ms / 2500–3000 ms), age (6.5 / 9.5 months), and their interactions as fixed effects. Subject-specific intercepts and by-subject condition slopes were included as random effects. Note, all *p*-values for fixed effects and their interactions were obtained here (i.e. for all linear and generalized linear mixed models used for analysis of EEG and behavioural data) using Type II Wald chi-square tests, and significant interactions were followed up by planned pairwise comparisons of least square means. Pairwise comparisons were Tukey-corrected for multiple comparisons, and for linear mixed models, degrees of freedom were approximated using the Kenward-Rogers method.

### 2.5 Coding of infant gaze

Infant eye movements were manually coded from videos recorded during the experiment by a researcher blind to the condition being presented/position and identity of congruent and incongruent actors. This enabled analysis of infant gaze to congruent versus incongruent actors in the preferential looking trials, as well as gaze behaviour in the different gaze following conditions. Videos were viewed in real-time and frame-by-frame to accurately identify onsets and offsets of infant eye movements (right, centre, left, off-screen/ambiguous). A second independent researcher coded a random 15% of preferential looking videos and 15% of gaze following videos at 6.5 and 9.5 months to establish inter-rater reliability. Excellent reliability was obtained for both the preferential looking trials (each age, *ĸ* > 0.84) and the gaze following conditions (each age, *ĸ* > 0.94).

### 2.6 Preparation and analysis of infant gaze data

The eyetrackingR package (Dink and Ferguson, 2015) was utilized for statistical analysis of the manually coded infant gaze data. We focused on all gaze samples directed towards the screen, and excluded any trials with excessive offscreen gaze time (defined as > 30% of the total trial time). For the preferential looking component, we investigated whether a bias towards looking at the congruent actor emerged between the baseline and test blocks. For the gaze following trials, we looked at gaze to the different AOIs (cued object/uncued object/adult face/adult target object) to explore differences in looking patterns between conditions. The AOIs were based on gaze to left/centre/right portions of the monitor on which the stimuli were presented.

#### 2.6.1 Infant gaze during preferential looking trials

A linear mixed model was used to explore differences in the mean congruent gaze bias during the baseline and test blocks (i.e. the proportion of time averaged across trials). The bias (or ‘preference’) was defined as the proportion of time within a trial spent looking at the congruent actor minus the proportion of time spent looking at the incongruent actor. The fixed effects were block (baseline/test) and age (6.5 m/9.5 m), as well as their interaction, and random effects included a subject-specific offset. To investigate whether differences emerged *within* trials (Schofield et al., 2013; Waxman et al., 2016), we also conducted two analyses. The first was a growth curve analysis (GCA) using mixed-effects models (Baayen et al., 2008; Mirman et al., 2008) to determine whether differences in the way looking to the congruent versus incongruent actor changed over the 5000 ms trial. To do this, we calculated (in 100 ms bins across the trial) the proportion of looking time that each infant devoted to the congruent actor minus the incongruent actor. This preference towards looking at the congruent actor was treated as the dependent measure, with block (baseline/test), age (6.5 months/9.5 months), orthogonal polynomial time codes (linear/quadratic/cubic/quartic growth trajectories), and their interactions included as fixed effects, and subject-specific intercepts included as random effects. The second analysis, to determine more precisely the timing and length of any significant differences identified by the GCA, was a bootstrapped smoothed divergence analysis with Bonferroni-correction (Wendt et al., 2014). This allowed us to estimate more precisely the times at which differences between blocks emerged, and for how long. Again, the bias towards looking to the congruent actor was used as the dependent measure.

#### 2.6.2 Infant gaze during gaze following trials

To explore any differences in infant’s own gaze during the three conditions (0 to 3000 ms after the onset of adult head turn), we performed the following analyses: i) a GCA to determine whether any differences in gaze emerged over the course of a trial, with AOI, condition, and age, as well as their interactions (and interactions with first through fourth order time polynomials) as fixed effects, and subject-specific intercepts and by-subject condition slopes as random effects. The dependent variable was the logit-transformed proportion looking time in order to avoid problems with analysing raw proportions with linear models (Jaeger, 2008; Waxman et al., 2016); ii) linear mixed models to investigate differences between conditions in the overall frequency of gaze shifts, and of the frequency or latency of different gaze-shift patterns (i.e. from cued object to face/face to adult target object/face to cued object/face to uncued object). These models included age, condition, and their interactions as fixed effects, and subject-specific offsets as random effects. The number of gaze shifts, logit-transformed proportion of trials, or latency of gaze shifts was treated as the dependent variable. Models using the number of gaze shifts (count data) as the dependent variable were generalized linear mixed models with Poisson family logit link functions.

## 3 Results

### 3.1 Main EEG analysis

To be included in the following analyses, infants were required to have a minimum of five trials per condition after pre-processing of the EEG data (Cannon et al., 2016; Marshall et al., 2011, 2013, Rayson et al., 2016, 2017). This left a total of 22 infants at 6.5 months and 19 infants at 9.5 months, with an average of 12.83 (SD = 4.11) trials per condition at 6.5 months (congruent, M = 12.86, SD = 4.54; incongruent, M = 12.59, SD = 4.73; scrambled, M = 13.05, SD = 4.26) and 14.63 (SD = 4.95) trials at 9.5 months (congruent, M = 14.95, SD = 5.69; incongruent, M = 14.74, SD = 5.25; scrambled, M = 14.21, SD = 4.49).

The linear mixed model analysis revealed main effects of both electrode cluster (χ^2^(7) = 89.51, *p* < 0.0001) and WOI (χ^2^(5) = 206.16, *p* < 0.0001); these were qualified by two-way interactions between age and cluster (χ^2^(7) = 44.29, *p* < 0.0001), age and condition (χ^2^(2) = 14.04, *p* < 0.001), condition and cluster (χ^2^(14) = 99.89, *p* < 0.0001), and cluster and WOI (χ^2^(35) = 73.77, *p* < 0.0005), as well as a three-way interaction between age, cluster, and condition (χ^2^(14) = 25.19, *p* = 0.033). Pairwise comparisons revealed that, at both ages, alpha ERD was greater in the congruent condition than the incongruent (6.5 m: *t*(135.31) = -2.63, *p* =  0.025; 9.5m: *t*(170.31) = -4.41, *p* <  0.001) and scrambled (6.5m: *t*(194.98) = -2.76, *p* = 0.017; 9.5 m: *t*(250.07) = -6.7, *p* <  0.0001) conditions in C4. In P4, alpha ERD was also stronger in the congruent versus incongruent condition at both ages (6.5m: *t*(135.31) = -2.56, *p* =  0.031; 9.5m: *t*(172.41) = -3.15, *p* =  0.006), and stronger in the congruent than scrambled condition at 9.5m (*t*(253.37) = -3.17, *p* =  0.005). At 9.5m this difference in conditions extended to P3 as well (congruent – incongruent: *t*(172.41) = -2.79, *p* =  0.016; congruent – scrambled: *t*(253.37) = -3.01; *p* =  0.008). In F3, there was significantly less alpha power in scrambled at 6.5m compared to 9.5m (*t*(5763.86) = -2.13, *p* =  0.033), with significant ERS in the congruent condition during the last time window (see Table S1 for comparisons of power to baseline at each age, electrode cluster, condition, and time period) at 9.5m (*t*(17) = 2.67; *p* =  0.016). From 6.5m to 9.5m, alpha power decreased in the scrambled condition in C3 (*t*(5762.79) = 3.37, *p* <  0.001) and F4 (*t*(5763.25) = 2.1, *p* =  0.036). In the congruent condition, alpha ERD increased from 6.5m to 9.5m in C4 (*t*(5765.07) = 3.79, *p* < 0.0005) and P3 (*t*(*5765.1*) = 4.59, *p* <  0.0001). Results from the WOI × cluster interaction follow-up comparisons can be found in Figure S2. Note, as hypothesized, no significant differences between conditions were revealed in occipital clusters.

**Fig. 3:**
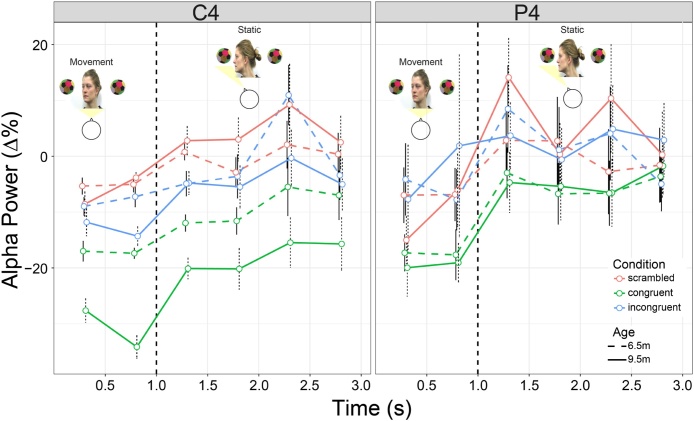
Baseline-corrected alpha power in C4 (left) and P4 (right) electrode clusters over time at 6.5 months (dashed line) and 9.5 months (solid line) during the scrambled (red), congruent (green), and incongruent (blue) conditions. Time zero is the start of the adult actor’s head turn. The vertical dashed lines indicate the end of the observed head turn, which was followed by a static period of adult gaze towards the object. Error bars represent +/− standard error (For interpretation of the references to colour in this figure legend, the reader is referred to the web version of this article).

**Fig. 4:**
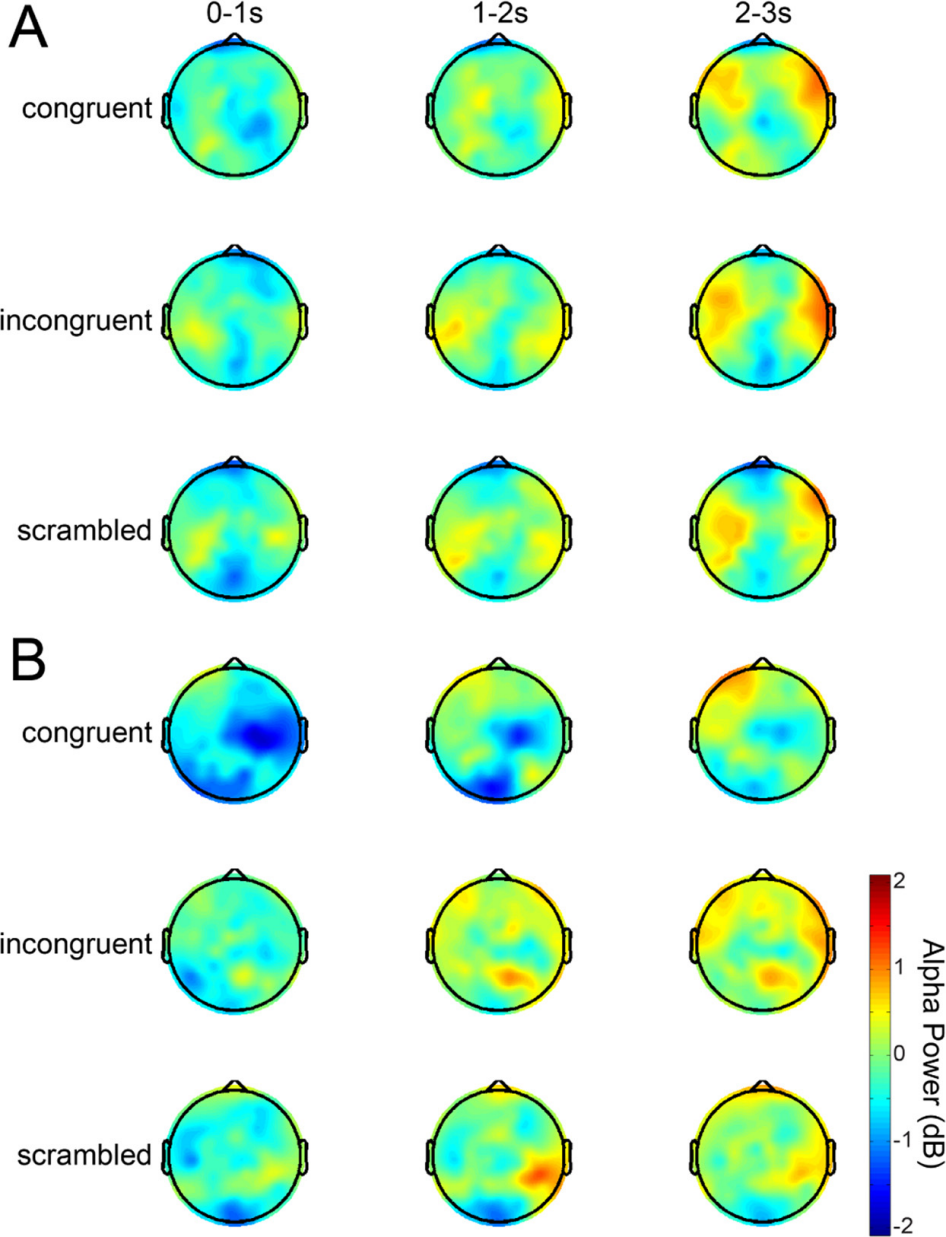
Scalp topographies of baseline-corrected alpha power in each condition at 6.5 months (A) and 9.5 months (B).

Findings in C4 and P4 (i.e. where alpha ERD was greater in congruent compared to incongruent and scrambled conditions at both ages) are illustrated in Fig. 3. Results from all clusters can be found in Figure S1, and scalp topographies of alpha power over all electrodes are shown in Fig. 4.

### 3.2 Infant gaze behaviour during EEG trials

**Fig. 5:**
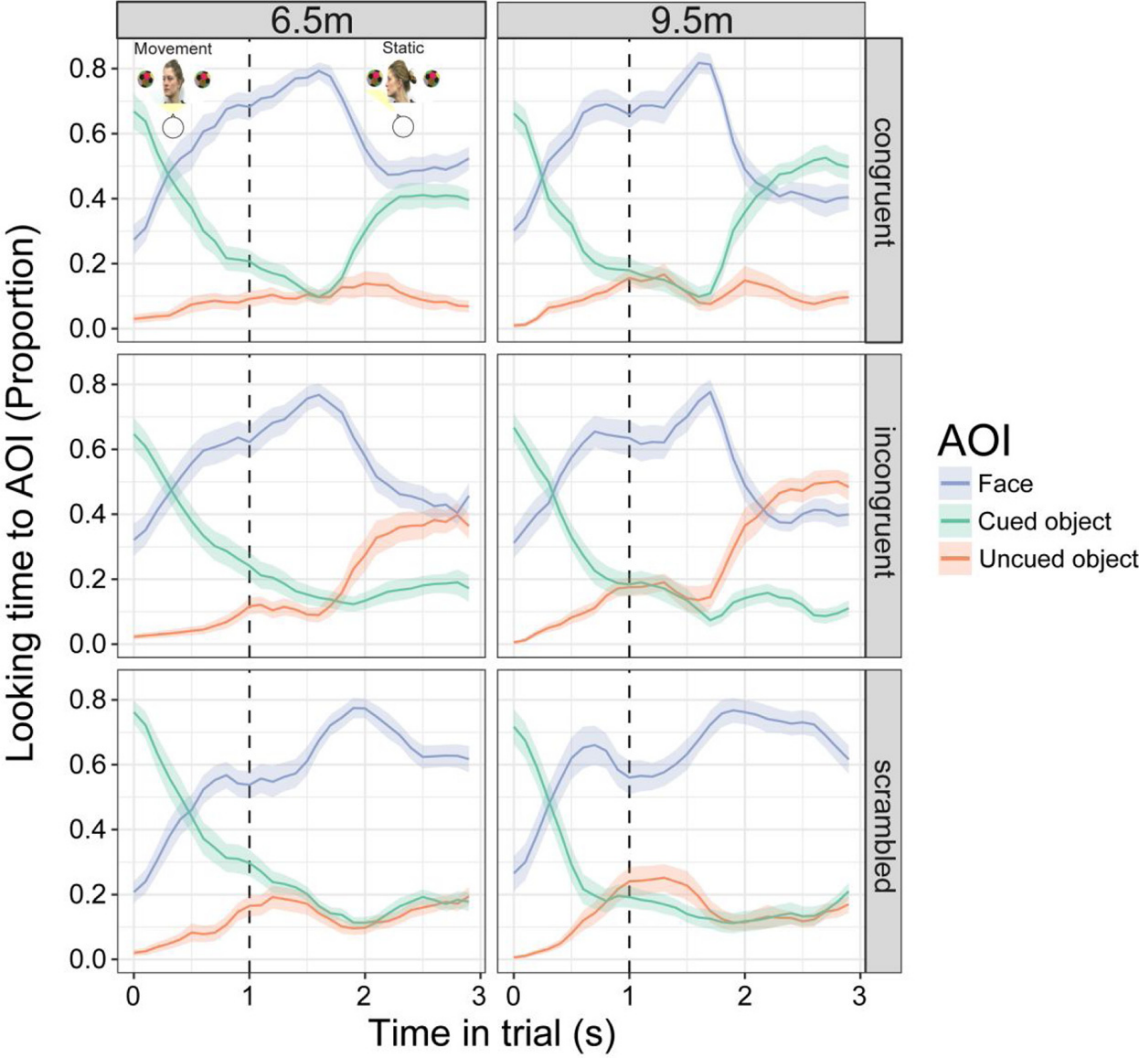
Time-course of looking to the different AOIs in the different conditions, at both ages. Time zero is the start of the adult actor’s head turn. The vertical dashed lines indicate the end of the observed head turn, which was followed by a static period of adult gaze towards the object. Shaded regions represent +/− standard error.

The time-course of infant gaze in the three gaze following conditions can be seen in Fig. 5 (i.e. the probability of looking to the cued object/adult face/uncued object over time; see Figure S4 for gaze time-courses split by AOI). Also, see Fig. 1 for typical gaze behaviour during different stages of the trial in the different conditions. Infants looked to the screen for the same proportion of time during trials in each condition (*χ^2^*(2) = 1.33, *p* = 0.515). Looking patterns were very similar in the congruent and incongruent conditions, with infants looking from the cued object to the adult’s face, then to the object the adult had turned towards regardless of whether it was previously cued or uncued. However, in the scrambled condition, although infants did look from the cued object to the scrambled face, they then continued to look there rather than follow the direction of coherent motion to an object. Indeed, the GCA analysis revealed a significant four-way interaction between AOI, condition, age, and the linear temporal function (*χ^2^*(4) = 61.82, *p* < 0.0001), with results from this analysis also suggesting that some differences emerged between 6.5 months and 9.5 months. Specifically, infants appeared even more likely to follow the adult’s gaze in both congruent and incongruent conditions by 9.5 months (separate cued object model: condition × age × ot1, *χ^2^*(2) = 41.22, *p* < 0 .0001; separate uncued object model: condition × age × ot1, *χ^2^*(2) = 21.92, *p* < 0.0001), and were even more likely to keep looking at the face in the scrambled condition (separate face model: condition × age × ot1, *χ^2^*(2) = 6.80, *p* = 0.033; see Fig. 5 and Figure S5).

Results from the other models run to look at the frequency or latency of gaze shifts/looking patterns during gaze following trials can be found in the SI (these results are in keeping with the above GCA results).

### 3.3 Scrambled gaze following trials: Congruent versus incongruent

We performed similar analyses to those above for alpha power and infant gaze during gaze following trials, but on scrambled trials split by congruency (i.e. congruent or incongruent coherent motion). Details concerning these analyses can be found in the SI. To summarise, alpha power in congruent scrambled trials was not lower than in incongruent scrambled trials in C4 or P4 (Figure S6). In fact, at 6.5 m, there was less alpha power in P4 during incongruent scrambled trials than congruent. Moreover, there was lower alpha power in congruent compared to incongruent scrambled trials in F3 at 6.5 m, but this diminished by 9.5 m (Figure S6). Infants looked to the screen the same amount in both conditions, and a GCA revealed no effects of congruency on infant gaze behaviour (Figures S7, S8).

### 3.4 Preferential looking

To be included in the following analyses, infants were required to observe a baseline and test block of preferential looking trials. This left a total of 21 infants at 6.5 months and 19 infants at 9.5 months. The linear mixed model with age (6.5/9.5 months) and preferential looking block (baseline/test) as fixed effects and bias score (i.e. time looking at congruent minus incongruent) as the dependent variable did not reveal any significant results. However, interesting differences were revealed by the GCA and divergence analyses.

**Fig. 6:**
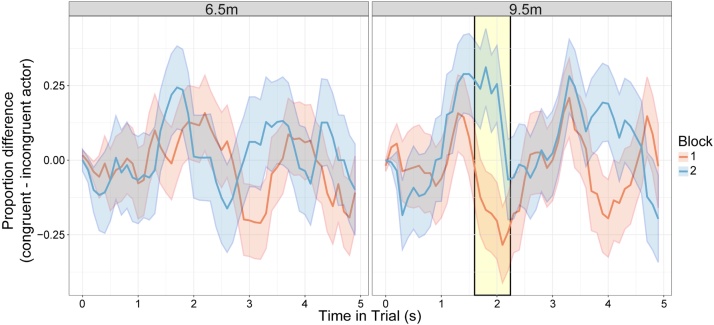
The time-course of gaze bias over the preferential gaze trials. This shows infants’ bias towards looking at the congruent actor (calculated as the difference between the proportion of time looking at the congruent actor minus the proportion of time looking at the incongruent actor). A proportions difference of 0.0 indicates equal gaze to the two actors, with a positive proportion difference indicating a congruent actor bias. The shaded regions around each line represent +/− standard error, and the yellow shaded region marks the segment at 9.5 months when infants' gaze in the two blocks significantly diverged (For interpretation of the references to colour in this figure legend, the reader is referred to the web version of this article).

The GCA revealed a significant three-way interaction between block, age, and the quadratic temporal function (*χ^2^*(1) = 6.77, *p* =  0.009). This interaction suggests that although no bias was apparent at 6.5 months, one did emerge by 9.5 months (Fig. 6 displays the continuous time-course of infants' looking bias to the congruent actor in the two blocks, at both 6.5 and 9.5 months of age). More specifically, this indicates that there was no bias towards the congruent actor in block 1 (the baseline period) at 9.5 months, but in block 2 (the test period) there was a significant bias towards looking at the congruent actor. That is, there was a significant rise-to and fall-from peak bias towards the congruent actor in block 2 but not in block 1. The subsequent divergence analysis showed that this difference appeared in the first half of the trial, with the two blocks diverging at around 1600 ms after stimulus onset, for around 600 ms. A divergence analysis was also conducted on the 6.5-month data, but as indicated by results from the GCA, no significant results were revealed.

### 3.5 Link between EEG and preferential looking

**Fig. 7:**
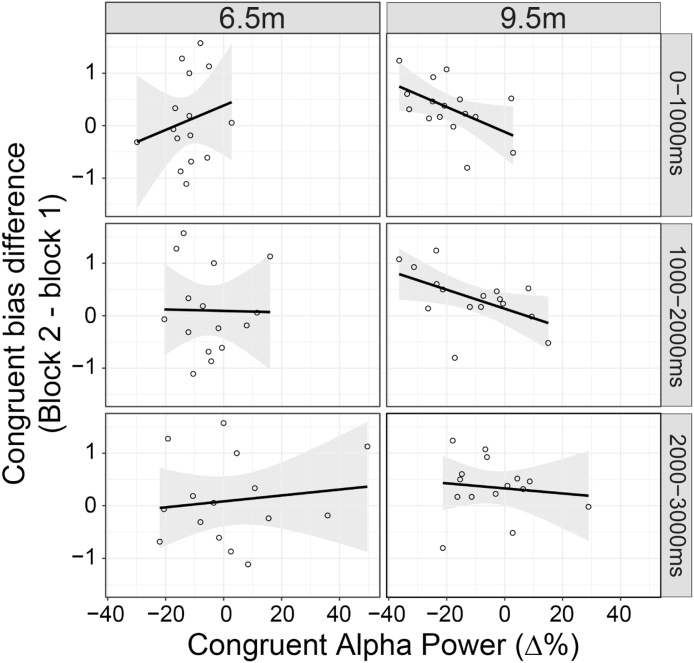
Relationship between baseline-corrected alpha power in clusters C4 and P4 during the congruent condition and the change in the congruent bias (congruent/incongruent bias in block 2 – congruent/incongruent bias in block 1) during the preferential looking trials at 6.5 months and 9.5 months. Shaded regions represent 95% confidence interval.

Finally, we examined whether individual differences in alpha power in the congruent gaze following condition at 9.5 months predicted the change in the degree of bias towards looking to the congruent actor (in the time period identified in the divergence analysis) in the preferential gaze trials at 9.5 months. A regression analysis revealed that alpha power during the congruent gaze following condition (averaged over C4 and P4; where congruent alpha ERD significantly differed from the other conditions at both ages) predicted the change in the congruent bias from baseline to test block (congruent bias in test block – congruent bias in baseline block; Fig. 7). Thus, the stronger the alpha ERD in the congruent condition, the more 9.5-month-old infants preferred to look at the congruent versus incongruent actor in the test block compared to the baseline block. This relationship (see Fig. 7) was found during the 1000 ms adult head turn (*t*(1,14)= −2.256, *p* =  0.041) and the 1000ms static period afterwards (*t*(1,14) = −2.338, *p* =  0.035), but not the last 1000ms of the static period (*t*(1,14) = −0.43, *p* =  0.674), by which time infants had generally followed the adult’s orientation to the target. No such relationships were found at 6.5 months.

## 4 Discussion

Results from this study advance our understanding of a critical, but little examined component of IJA in early infancy: detecting when one’s own gaze has been followed. Findings confirm that even in the first months of life, infants are sensitive to an adult following their gaze. As predicted, this sensitivity increases between 6.5 and 9.5 months, evidenced neurally by the enhancement of alpha ERD, and behaviourally by the emergence of a bias towards looking at an adult who had previously followed the infant’s gaze.

More specifically, infants here observed two adults shifting their gaze in a congruent or incongruent manner, based on the infant’s prior gaze shift. Infants also observed a control condition consisting of scrambled versions of the adult gaze shifts, with coherent motion congruent or incongruent to their previous shift. Regardless of age, more alpha ERD occurred in right central and parietal electrode clusters in the congruent compared to incongruent and scrambled conditions. Changes between 6.5 and 9.5 months included the strengthening of alpha ERD in central and parietal clusters, more ERD overall in the congruent compared to incongruent and scrambled conditions, and greater ERD during observation of congruent gaze shifts specifically. Moreover, after observing a number of gaze following trials, 9.5-month-olds demonstrated a preference for looking to the congruent versus incongruent actor. The degree of preference at this age was predicted by the magnitude of alpha ERD in the congruent gaze following condition, with more ERD in right centro-parietal electrodes related to a stronger gaze bias. Infant gaze to the adult’s face and the two objects was very similar during congruent and incongruent trials: infants tended to look from the cued object to the adult’s face, and then follow the adult’s gaze regardless of its congruency. Importantly, therefore, it is unlikely that differences in alpha ERD between these two conditions were driven simply by differences in the number of infant gaze shifts per trial, especially at the earliest time periods analysed.

The scrambled control condition was created in order to preserve low-level visual features from the original videos such as global coherent motion, while at the same time eliminating recognizable faces. Infants are sensitive to global coherent motion in random dot kinematograms in the first 2 months of life (Banton and Bertenthal, 1996; Wattam-Bell, 1994, 1992), and by 3 months of age, motion coherence discrimination thresholds are already around 50% (the percentage of dots moving in the same direction required for accurate discrimination; Wattam-Bell, 1994). Our scrambled condition was analogous to a random dot kinematogram with very high motion coherence (all stimuli above 60% during the head turn). During observation of the scrambled stimuli, differences from the congruent actor condition were apparent from the very earliest time periods, even though infants did not tend to follow the direction of motion after looking from the cued object to the adults’ face. This remained the case when scrambled stimuli were themselves split into congruent and incongruent conditions. Additionally, the effect of congruency revealed during observation of unscrambled stimuli on alpha ERD in central and parietal clusters was not present for congruent versus incongruent scrambled motion when these trials were split, suggesting that the effect was specific to the observation of an actor’s congruent gaze shift rather than congruent motion of an arbitrary stimulus.

### 4.1 Functional significance of the alpha band

Attenuation of power in the alpha band has been linked to a number of processes and the recruitment of various brain regions (Bell, 2002; Cannon et al., 2016; Klimesch, 1999; Pineda, 2005; Rayson et al., 2016), and probably varies in its functional significance between tasks and/or at different stages of processing (Xie et al., 2018). As the effect of congruency on ERD here was in central and parietal electrode clusters only, this finding can be interpreted in terms of two functional roles that are often attributed to alpha power decreases in electrodes over these areas: attention and action-perception matching.

A reduction in alpha power over parieto-occipital regions (‘visual’ alpha) has been linked specifically to attentional or arousal mechanisms, such as the suppression of irrelevant environmental information (Michel et al., 2015; Ward, 2003). Widespread alpha ERD has been observed in 9-month-olds who are currently looking at the same object as an adult, after that adult has made eye contact with them (Hoehl et al., 2014), and infant alpha ERD is greater during observation of another’s object-directed compared to object-averted gaze (Michel et al., 2015). Alpha ERD in similar regions is also found in adults during concurrent gaze to an object in the context of JA (Lachat et al., 2012). All these studies have linked parieto-occipital alpha to increases in attention, and hence, to processes that could aid object encoding and social learning in infancy (Hoehl et al., 2014; Michel et al., 2015). Although actors in both conditions made eye contact with the infant before turning to an object in our study, differences between alpha ERD in congruent and incongruent trials may have reflected, at least in part, more focused attention in the congruent condition. For example, only in the congruent condition did infants gaze at the same object twice (first when cued, then after following the adult). In the incongruent condition, infants followed the adult’s gaze to an object that they had not previously looked at, and in the scrambled condition infants tended to fixate only on the scrambled face itself. Hence, it is possible that the difference in alpha ERD between conditions was driven by an attentional overlap in the congruent condition, with greater attentional resources dedicated to the processing of the object and/or congruent actor (Striano et al., 2006).

Alpha activity in centro-parietal regions, often referred to as the ‘mu’ rhythm, is associated with the processing of social stimuli and recognition of others’ actions (Muthukumaraswamy et al., 2004; Oberman et al., 2007; Perry et al., 2011). Mu ERD is considered an index of sensorimotor system activity (Thorpe et al., 2016), and occurs during both execution and observation of similar actions (Vanderwert et al., 2013). Accordingly, mu ERD is widely used as a proxy measure of an action-perception matching (or ‘mirror’) mechanism, which maps between the visual and motor representations of actions (Fox et al., 2016; Marshall and Meltzoff, 2011). In our study, it is possible that such a mechanism aided infants in matching the congruent adult’s gaze shift to their own. Although typically associated with the encoding of manual actions or facial gestures (di Pellegrino et al., 1992; Ferrari et al., 2003), mirror-like neurons for attention orienting and head rotation have been found in macaque monkeys (Lanzilotto et al., 2017; Shepherd et al., 2009), and observed gaze direction modulates the activity of premotor mirror neurons selective for grasping (Coude et al., 2016). Furthermore, mu ERD has been observed in adults during both RJA and IJA (Lachat et al., 2012), suggesting that JA may involve a mechanism of attention mirroring (Lachat et al., 2012; Shepherd et al., 2009; Triesch et al., 2007). This possibility is supported by an additional analysis we conducted on alpha power during infants’ own head turns (see SI and Figure S5), with significant ERD revealed during execution as well observation of head turns. However, while adult actors in the stimuli used here performed head turns to gaze at the objects, infants tended to move only their eyes when performing gaze shifts. This was likely due to the experimental set-up, with a small visual angle between objects on the screen. As such, if an action-perception mechanism was implicated here, matching must have been partial, and/or have occurred at some level other than kinematics at which head and eye movements can be compared. One possibility is that these actions are represented in terms of their goal, e.g. the object, location, or direction that the motor act is aimed towards, and indeed, infants do appear to represent human actions as object-directed from early on in the first year of life (Sommerville et al., 2005; Woodward, 1998; Woodward and Gerson, 2014).

Interestingly, alpha ERD occurred even in the earliest time period we analysed, before the adult’s head turn had been completed. As one adult actor always followed the infant’s gaze and the other actor always looked in the opposite direction, infants were able to learn which actor would follow their gaze and which actor would not (see discussion of preferential looking results in the next section). As such, the greater alpha ERD early on during the congruent condition could reflect the prediction or anticipation of a *matching* adult response, given the identity of the adult actor (Denis et al., 2017; Saby et al., 2012; Southgate et al., 2009). Importantly, the adult’s response in both the congruent and incongruent conditions was contingent on the infant’s behaviour and was consistent (i.e. one actor always responded congruently and the other always responded incongruently), therefore both conditions were equally predictable. This claim is supported by an analysis of fronto-medial theta power (SI), which increases when expectations are violated (e.g. Berger et al., 2006; Conejero et al., 2018). We found no differences in theta power between the incongruent and congruent conditions. Any differences in alpha ERD between these two conditions were therefore unlikely to be driven by predictability per se, but instead, were influenced by whether this prediction matched the infant’s previous gaze shift. Such prediction could be achieved using a generative, or forward, model as suggested by some mirror system accounts (Kilner et al., 2009; Rizzolatti et al., 2014). Alternatively, infants could have identified the action outcome or goal, prior to sensorimotor activation, with any ensuing sensorimotor activity resulting from an attempt to emulate how the ongoing action would unfold using an inverse model (Csibra, 2007). In either case, the ability to predict others’ actions seems to improve over the latter half of the first year (Gredebäck et al., 2018), which corresponds to the timeline of change we observed between 6.5 and 9.5 months of age.

Based on previous findings regarding infant neural responses to having their gaze followed (Grossmann et al., 2013), differences in left prefrontal regions were expected here. Although we did not find a significant difference between conditions in this area, we did find significant alpha ERS relative to baseline in the left frontal cluster of electrodes at 9.5 months during the last time-period, in the congruent condition only (see Figure S1 and Table S1). Grossmann et al. (Grossmann et al., 2013) similarly did not find a significant difference between conditions, but only a change from baseline during congruent gaze. One possibility is that the activity in this particular period, in this region, reflected a match between the object that the adult actor is currently attending to and the infant’s working memory trace of their own initial gaze to this object. In support of this, frontal regions are commonly implicated in working memory in infants (Baird et al., 2002), as well as older children and adults (Crone et al., 2006; Kane and Engle, 2002; Nee et al., 2013), and alpha ERS in frontal areas has been associated with working memory processes at 8 months of age (Bell, 2002, 2001).

### 4.2 Preference for gaze followers and links to reward

As noted in the introduction, having one’s own gaze followed in adulthood recruits reward-related brain regions (Schilbach et al., 2010) and influences how one responds to a social partner (Bayliss et al., 2013; Grynszpan et al., 2017; Willemse et al., 2018). This is reminiscent of social imitation or mimicry, for example, having one’s own facial expressions imitated (Chartrand and Bargh, 1999; Edwards et al., 2015; Hoffman et al., 2006; Hove and Risen, 2009; Lachat et al., 2012; Shepherd et al., 2009; Triesch et al., 2007). Our finding that 9.5 month old infants demonstrated a gaze bias towards the congruent versus incongruent actor is in keeping with previous research showing that infants prefer looking at an adult who imitates them (Agnetta and Rochat, 2004; Meltzoff, 1990). Interestingly, even monkeys demonstrate a comparable gaze bias towards an experimenter who matches their behaviour in early infancy (Sclafani et al., 2015; Simpson et al., 2014). Being imitated may increase an infant’s awareness of another’s attention to their experience (Meltzoff, 2007, 1995; Mundy, 2018; Reddy, 2003), and thus could be indicative to the infant of the other’s prosocial stance towards them (Powell and Spelke, 2018). This is hypothesized to play an important role in JA development (Mundy, 2018). The experience of having one’s own gaze followed therefore appears similar to that of other forms of imitation in early infancy, with greater reward value possibly assigned to the congruent adult (Neufeld and Chakrabarti, 2016).

The difference in alpha ERD between congruent and incongruent conditions is also in keeping with the involvement of reward mechanisms in the detection of having one’s own gaze followed. The mu rhythm has been linked to reward in adults (Brown et al., 2013; Trilla Gros et al., 2015), where social stimuli associated with a greater reward value are related to stronger mu ERD during observation. This relationship has been localized to the right hemisphere (Trilla Gros et al., 2015), again corresponding with our results. In addition, the EEG response we found is similar to that seen in slightly older infants when an adult imitates them (Reid et al., 2011; Saby et al., 2012), which is around the same age at which infants demonstrate a bias towards looking at imitators (Agnetta and Rochat, 2004; Meltzoff, 1996, 1990). Intriguingly, the degree of predictive sensorimotor activity during action observation is modulated by social relevance (Kilner et al., 2006; Perry et al., 2011) and the extent to which participants perceive the observed individual as an interactive partner (Kourtis et al., 2010), which here, could have been increased by the ‘imitation’ of infants’ gaze shifts in the congruent condition.

### 4.3 Future directions

It is interesting that a neural response to others’ gaze following was revealed at 6.5 months, but preferential gaze to the congruent actor was only found at 9.5 months. Although infants may be sensitive to having their gaze followed at a neural level from an earlier age, further refinement of the mechanisms involved and coordination with other neural circuits that are still developing may be required for demonstration of a gaze bias later on. This improvement in the ability to detect others’ gaze shifts does fit with the timeline of JA emergence, with much improvement in JA-related skills from 6 months (Mundy et al., 2007), and demonstration of clear IJA-like behaviours not apparent until 10–12 months of age (Beuker et al., 2013; Carpenter et al., 1998; Mundy et al., 2007). Further research is now required to explore this relationship, as well as the specific relationship and cognitive transitions between an early sensitivity to others’ gaze following and later IJA skill.

Another interesting direction for future studies is to look at how early social experience might relate to the neural and behavioural effects we identified here. The degree to which mothers imitate their infants’ behaviour during early social interactions is related an infant’s ability to detect and respond to reciprocal interactions (Bigelow and Walden, 2009), the emergence of infant social expressiveness (Murray et al., 2016), and the strength of mu ERD during observation of adult facial expressions (Rayson et al., 2017). Observational work also suggests that the degree to which mothers follow rather than direct infants during early interactions is related to IJA emergence (Gaffan et al., 2010), and one computational model implicates early mother-infant interactions in the development of an action-perception matching mechanism for gaze (Triesch et al., 2007). Early social interactions may therefore play an important role in the development of the capacity to detect that one’s own gaze has been followed.

### 4.4 Conclusion

Vital for recognition of whether an attempt to direct someone else’s attention has been successful, and thus whether or not a triadic interaction state has been achieved, detecting that one’s own gaze has been followed represents a fundamental building block of IJA. Our study confirms that infants are sensitive to someone following their gaze from an early age, with improvements in this capacity occurring between 6.5 and 9.5 months at a neural and behavioural level. The pattern of alpha attenuation revealed here likely reflects the recruitment and critical development of several brain networks including those linked to attention, action-perception matching, reward, and working memory processes, which must be functionally connected in order to coordinate the complex processing and motor responses required for true IJA. An important challenge for future research is to elucidate the factors that contribute to the emergence of a capacity to detect others’ gaze following, as well as to investigate more explicitly how this relates to the emergence of IJA. This will increase understanding of the precursors and early development of JA, and thus how this may impact other skills critical for social functioning in later childhood.

## Funding

This work was supported by a Medical Research Council UK doctoral studentship (MR/J003980/1) awarded to Holly Rayson.

## Conflict of interest statement

The authors declare no conflict of interest.
