# Supplementary material for: Building blocks of joint attention: Early sensitivity to having one’s own gaze followed
Source: Dev Cogn Neurosci. 2019 Mar 5;37:100631. doi: 10.1016/j.dcn.2019.100631 (PMC6556871; doi:10.1016/j.dcn.2019.100631)
Supplement: Supplementary file 1 [file mmc1.docx]

**SUPPLEMENTARY INFORMATION**

**METHODS**

***Participant exclusions***

In total, 38 infants (22 male, 16 female) aged approximately 6.5 months took part in this experiment, with 34 of the same infants (19 male, 15 female) also participating at 9.5 months of age. 15 infants at 6.5 months, and 10 infants at 9.5 months, were excluded prior to EEG analysis due to fussiness during net placement or throughout the experiment (6.5 months, N = 14; 9.5 months, N = 8), or technical difficulties (6.5 months, N = 1; 9.5 months, N = 2). This left a total of 23 infants at 6.5 months and 24 infants at 9.5 months. This loss of data is comparable with other infant EEG studies (e.g. Cannon et al., 2016; Marshall et al., 2013; Rayson et al., 2017; Southgate et al., 2009).

***Scrambled stimuli***

For the control condition, scrambled versions of each video were produced by dividing the face region into square blocks (18 × 18 pixels), randomly shuffling these blocks in the first frame of the video and then applying the same transformation to each subsequent frame. The motion coherence of each video was computed by calculating optical flow in each 18 × 18 pixel block (Farneback, 2000), and then computing the percentage of blocks whose motion direction fell within 15° of the peak motion direction over all frames. The resulting motion coherence value was over 60% for all videos (scrambled and unscrambled) during the period of the head turn. This process thus preserved coherent motion in the same direction as the head turn in the original video (with most 18 × 18 pixel blocks containing either no motion or motion to the left or right, depending on the trial), and resulted in a video with similar low-level visual and motion features as the original, but without higher order visual features that support recognition of the stimulus as a face. 10 adults were asked to view these stimuli and judge the direction of motion (left/right/ambiguous) in the scrambled videos. The direction was correctly identified in 100% of cases.

***Gaze cueing to highlighted object***

The proportions of trials where infants’ gaze was successfully drawn to the cued object are as follows: 6.5m congruent, M = 0.86 (SD = 0.16); 6.5m incongruent, M = 0.82 (SD = 0.20); 6.5m scrambled, M = 0.87 (SD = 0.16); 9.5m congruent, M = 0.88 (SD = 0.08); 9.5m incongruent, M = 0.84 (SD=0.12); 9.5m scrambled, M = 0.89 (SD = 0.06). Note, a linear mixed model analysis was conducted to compare these proportions (logit-adjusted), which included age (6.5m/9.5m), condition (congruent/incongruent/scrambled), and their interactions as fixed effects, and a subject-specific intercept as a random effect. This revealed no significant main effects or interactions.

***Pre-processing of EEG data***

After viewing the video recordings and marking periods of inattention/movement using EGI software (NetStation v4.3.1; Electrical Geodesics, Inc., Eugene, OR), EEG data were exported and analysed using the EEGLAB v13.3.2 toolbox (Delorme and Makeig, 2004). The PREP pipeline toolbox was used to identify and interpolate noisy channels, as well as to apply robust average re-referencing (Bigdely-Shamlo et al., 2015). Data were then bandpass filtered at 2-35 Hz. A natural-gradient logistic infomax independent component analysis (ICA) was performed on the data (the runica algorithm; Delorme and Makeig, 2004) to decompose the EEG mixed signals into their underlying neural and artefactual components (such as eye and muscle movements). Artefact components were identified and removed using the ADJUST algorithm (v1.1; Mognon et al., 2011). Epochs ranging from 300ms before to 5800ms after the appearance of the static face were extracted. Epochs that contained previously marked periods of inattention/movement (including head turns, which were analysed separately as an execution condition) and epochs in which more than 15% of channels exceeded +/- 250µV were excluded.

**RESULTS**

***Infant movement during each condition***

In order to rule out the possibility that differences in infant movement could drive any potential differences in alpha activity, we compared the number of artefactual movements (i.e. *not* saccades or head turns) and the number of movements linked to gaze shifts (saccades or head turns) using a generalized linear mixed model with a Poisson family logit link function. The number of movements was used as the dependent variable, with age, condition, and their interaction included as fixed effects, as well as subject-specific intercepts and by-subject condition slopes as random effects. There was no main effect of age or condition, nor was there an age × condition interaction effect on the number of artefactual movements. There was a main effect of age on gaze shift movements (*χ^2^*(1) = 17.32, *p* < 0.0001), with infants making more gaze shift movements at 9.5m than 6.5m (*Z* = -2.12, *p* = 0.034). To exclude the possibility that small, un-identified movements could have induced channel artefacts resulting in differential numbers of trials being rejected between conditions at the pre-processing stage, we similarly used a generalized linear mixed model with Poisson family logit link function to compare the number of epochs in the final analysis using age, condition, and their interaction as fixed effects, and subject-specific intercepts and by-subject condition slopes as random effects. There was no effect of age or condition on the number of epochs included in the final analysis.

***Alpha activity during observation of the three gaze following conditions***

Significant differences in alpha power compared to baseline during the different conditions are listed in Table S1. Results from all electrode clusters included in the analyses are illustrated in Figure S1, significant differences between time windows of interest (WOIs) are shown in Figure S2, and spectrograms of activity in C4 are shown in Figure S3.

**Table S1:** Percentage change in alpha power compared to baseline during observation of the three gaze following conditions. Negative values reflect a reduction in power (event-related desynchronization; ERD) and positive values reflect an increase in power (event-related synchronization; ERS). Statistically significant differences are denoted by asterisks (* p < 0.05, ** p < 0.01, *** p < 0.001).

| ***Cluster*** | ***Age*** | ***Condition*** | ***WOI*** | ***Alpha Power (Δ%)*** | ***Statistic*** |
| --- | --- | --- | --- | --- | --- |
| F3 | 6m | congruent | 0-500ms | -5.96 | *t*(21) = -1.06, *p* = 0.303 |
|  |  |  | 500-1000ms | -16.68 | *t*(21) = 4.65, *p* < 0.001** |
|  |  |  | 1000-1500ms | -5.61 | *t*(21) = -0.87, *p* = 0.392 |
|  |  |  | 1500-2000ms | -2.08 | *t*(21) = -0.23, *p* = 0.819 |
|  |  |  | 2000-2500ms | 11.94 | *t*(21) = 1.02, *p* = 0.318 |
|  |  |  | 2500-3000ms | 8.78 | *t*(21) = 1.16, *p* = 0.260 |
|  |  | incongruent | 0-500ms | -7.81 | *t*(21) = -1.64, *p* = 0.117 |
|  |  |  | 500-1000ms | -16.75 | *t*(21) = -4.09, *p* < 0.001** |
|  |  |  | 1000-1500ms | -7.93 | *t*(21) = -1.70, *p* = 0.103 |
|  |  |  | 1500-2000ms | -8.31 | *t*(21) = -1.51, *p* = 0.147 |
|  |  |  | 2000-2500ms | 4.81 | *t*(21) = 1.04, *p* = 0.310 |
|  |  |  | 2500-3000ms | 4.29 | *t*(21) = 0.69, *p* = 0.498 |
|  |  | scrambled | 0-500ms | -9.43 | *t*(21) = -1.77, *p* = 0.092 |
|  |  |  | 500-1000ms | -17.83 | *t*(21) = -4.83, *p* < 0.001** |
|  |  |  | 1000-1500ms | -9.25 | *t*(21) = -1.80, *p* = 0.087 |
|  |  |  | 1500-2000ms | -11.94 | *t*(21) = -2.19, *p* = 0.040* |
|  |  |  | 2000-2500ms | -12.28 | *t*(21) = -2.45, *p* = 0.023* |
|  |  |  | 2500-3000ms | 0.03 | *t*(21) = 0.00, *p* = 0.997 |
|  | 9m | congruent | 0-500ms | -7.35 | *t*(18) = -1.95, *p* = 0.067 |
|  |  |  | 500-1000ms | -11.79 | *t*(18) = -2.77, *p* = 0.013* |
|  |  |  | 1000-1500ms | 1.16 | *t*(17) = 0.18, *p* = 0.857 |
|  |  |  | 1500-2000ms | 4.64 | *t*(18) = 0.66, *p* = 0.519 |
|  |  |  | 2000-2500ms | 14.25 | *t*(17) = 1.71, *p* = 0.106 |
|  |  |  | 2500-3000ms | 19.41 | *t*(17) = 2.67, *p* = 0.016* |
|  |  | incongruent | 0-500ms | -12.27 | *t*(18) = -2.41, *p* = 0.027* |
|  |  |  | 500-1000ms | -10.15 | *t*(18) = -1.95, *p* = 0.067 |
|  |  |  | 1000-1500ms | -0.08 | *t*(17) = -0.01, *p* = 0.993 |
|  |  |  | 1500-2000ms | 6.91 | *t*(18) = 0.54, *p* = 0.593 |
|  |  |  | 2000-2500ms | 11.28 | *t*(17) = 1.15, *p* = 0.265 |
|  |  |  | 2500-3000ms | 6.52 | *t*(17) = 0.80, *p* = 0.434 |
|  |  | scrambled | 0-500ms | -10.61 | *t*(18) = -1.59, *p* = 0.130 |
|  |  |  | 500-1000ms | -12.19 | *t*(18) = -1.82, *p* = 0.085 |
|  |  |  | 1000-1500ms | -8.05 | *t*(17) = -1.05, *p* = 0.307 |
|  |  |  | 1500-2000ms | -1.43 | *t*(18) = -0.19, *p* = 0.849 |
|  |  |  | 2000-2500ms | 9.12 | *t*(17) = 0.66, *p* = 0.516 |
|  |  |  | 2500-3000ms | -4.19 | *t*(17) = -0.42, *p* = 0.676 |
| F4 | 6m | congruent | 0-500ms | -5.71 | *t*(21) = -0.74, *p* = 0.470 |
|  |  |  | 500-1000ms | -15.13 | *t*(21) = -2.38, *p* = 0.027* |
|  |  |  | 1000-1500ms | -9.69 | *t*(21) = -1.28, *p* = 0.214 |
|  |  |  | 1500-2000ms | -7.49 | *t*(21) = -1.25, *p* = 0.225 |
|  |  |  | 2000-2500ms | 1.29 | *t*(21) = 0.14, *p* = 0.892 |
|  |  |  | 2500-3000ms | 7.10 | *t*(21) = 1.04, *p* = 0.308 |
|  |  | incongruent | 0-500ms | -7.25 | *t*(21) = -1.30, *p* = 0.209 |
|  |  |  | 500-1000ms | -16.23 | *t*(21) = -3.63, *p* = 0.002** |
|  |  |  | 1000-1500ms | -3.97 | *t*(21) = -0.56, *p* = 0.583 |
|  |  |  | 1500-2000ms | -8.93 | *t*(21) = -1.81, *p* = 0.084 |
|  |  |  | 2000-2500ms | -2.75 | *t*(21) = -0.63, *p* = 0.534 |
|  |  |  | 2500-3000ms | -3.85 | *t*(21) = -0.67, *p* = 0.511 |
|  |  | scrambled | 0-500ms | -12.20 | *t*(21) = -2.93, *p* = 0.008** |
|  |  |  | 500-1000ms | -12.03 | *t*(21) = -2.11, *p* = 0.047* |
|  |  |  | 1000-1500ms | -3.39 | *t*(21) = -0.70, *p* = 0.489 |
|  |  |  | 1500-2000ms | 1.82 | *t*(21) = 0.34, *p* = 0.741 |
|  |  |  | 2000-2500ms | 1.99 | *t*(21) = 0.31, *p* = 0.758 |
|  |  |  | 2500-3000ms | 5.20 | *t*(21) = 0.61, *p* = 0.552 |
|  | 9m | congruent | 0-500ms | -14.78 | *t*(17) = -3.16, *p* = 0.006** |
|  |  |  | 500-1000ms | -21.63 | *t*(17) = -5.07, *p* < 0.001*** |
|  |  |  | 1000-1500ms | -4.15 | *t*(17) = -0.65, *p* = 0.526 |
|  |  |  | 1500-2000ms | -6.17 | *t*(18) = -1.31, *p* = 0.208 |
|  |  |  | 2000-2500ms | -4.93 | *t*(17) = -1.17, *p* = 0.258 |
|  |  |  | 2500-3000ms | -3.26 | *t*(17) = -0.55, *p* = 0.588 |
|  |  | incongruent | 0-500ms | -7.33 | *t*(17) = -1.43, *p* = 0.172 |
|  |  |  | 500-1000ms | -16.81 | *t*(17) = -3.86, *p* = 0.001** |
|  |  |  | 1000-1500ms | -1.89 | *t*(17) = -0.31, *p* = 0.761 |
|  |  |  | 1500-2000ms | 4.44 | *t*(18) = 0.73, *p* = 0.472 |
|  |  |  | 2000-2500ms | 1.83 | *t*(17) = 0.31, *p* = 0.762 |
|  |  |  | 2500-3000ms | 1.97 | *t*(17) = 0.24, *p* = 0.816 |
|  |  | scrambled | 0-500ms | -11.57 | *t*(17) = -2.96, *p* = 0.009** |
|  |  |  | 500-1000ms | -17.42 | *t*(17) = -4.49, *p* < 0.001*** |
|  |  |  | 1000-1500ms | -10.99 | *t*(17) = -1.96, *p* = 0.066 |
|  |  |  | 1500-2000ms | -7.92 | *t*(18) = -1.52, *p* = 0.147 |
|  |  |  | 2000-2500ms | -5.51 | *t*(17) = -1.00, *p* = 0.334 |
|  |  |  | 2500-3000ms | -9.31 | *t*(17) = -1.47, *p* = 0.159 |
| C3 | 6m | congruent | 0-500ms | -10.21 | *t*(21) = -1.96, *p* = 0.064 |
|  |  |  | 500-1000ms | -7.77 | *t*(21) = -1.76, *p* = 0.093 |
|  |  |  | 1000-1500ms | 0.86 | *t*(21) = 0.12, *p* = 0.909 |
|  |  |  | 1500-2000ms | -4.06 | *t*(21) = -0.69, *p* = 0.496 |
|  |  |  | 2000-2500ms | 3.70 | *t*(21) = 0.59, *p* = 0.563 |
|  |  |  | 2500-3000ms | -1.23 | *t*(21) = -0.20, *p* = 0.841 |
|  |  | incongruent | 0-500ms | -9.75 | *t*(21) = -1.61, *p* = 0.123 |
|  |  |  | 500-1000ms | -4.89 | *t*(21) = -0.81, *p* = 0.427 |
|  |  |  | 1000-1500ms | 0.22 | *t*(21) = 0.02, *p* = 0.981 |
|  |  |  | 1500-2000ms | 0.30 | *t*(21) = 0.03, *p* = 0.975 |
|  |  |  | 2000-2500ms | 3.15 | *t*(21) = 0.56, *p* = 0.581 |
|  |  |  | 2500-3000ms | -5.31 | *t*(21) = -0.99, *p* = 0.331 |
|  |  | scrambled | 0-500ms | -3.91 | *t*(21) = -0.92, *p* = 0.370 |
|  |  |  | 500-1000ms | -3.29 | *t*(21) = -0.72, *p* = 0.477 |
|  |  |  | 1000-1500ms | 3.04 | *t*(21) = 0.69, *p* = 0.497 |
|  |  |  | 1500-2000ms | 4.23 | *t*(21) = 1.25, *p* = 0.224 |
|  |  |  | 2000-2500ms | 7.04 | *t*(21) = 1.24, *p* = 0.230 |
|  |  |  | 2500-3000ms | 7.97 | *t*(21) = 1.33, *p* = 0.199 |
|  | 9m | congruent | 0-500ms | -13.76 | *t*(17) = -2.86, *p* = 0.011* |
|  |  |  | 500-1000ms | -15.35 | *t*(17) = -3.36, *p* = 0.004** |
|  |  |  | 1000-1500ms | -3.80 | *t*(17) = -0.52, *p* = 0.607 |
|  |  |  | 1500-2000ms | 1.57 | *t*(17) = 0.20, *p* = 0.847 |
|  |  |  | 2000-2500ms | 0.56 | *t*(17) = 0.07, *p* = 0.944 |
|  |  |  | 2500-3000ms | 1.47 | *t*(17) = 0.20, *p* = 0.846 |
|  |  | incongruent | 0-500ms | -8.46 | *t*(17) = -1.26, *p* = 0.226 |
|  |  |  | 500-1000ms | -10.53 | *t*(17) = -1.73, *p* = 0.102 |
|  |  |  | 1000-1500ms | -7.73 | *t*(17) = -1.14, *p* = 0.271 |
|  |  |  | 1500-2000ms | -1.51 | *t*(17) = -0.24, *p* = 0.810 |
|  |  |  | 2000-2500ms | -2.74 | *t*(17) = -0.46, *p* = 0.652 |
|  |  |  | 2500-3000ms | -6.41 | *t*(17) = -1.03, *p* = 0.318 |
|  |  | scrambled | 0-500ms | -17.03 | *t*(17) = -4.27, *p* < 0.001 |
|  |  |  | 500-1000ms | -11.69 | *t*(17) = -2.36, *p* = 0.031* |
|  |  |  | 1000-1500ms | -11.25 | *t*(17) = -1.61, *p* = 0.126 |
|  |  |  | 1500-2000ms | -3.14 | *t*(17) = -0.51, *p* = 0.613 |
|  |  |  | 2000-2500ms | -4.02 | *t*(17) = -0.46, *p* = 0.651 |
|  |  |  | 2500-3000ms | -5.50 | *t*(17) = -0.77, *p* = 0.451 |
| C4 | 6m | congruent | 0-500ms | -17.00 | *t*(21) = -9.54, *p* < 0.001*** |
|  |  |  | 500-1000ms | -17.38 | *t*(21) = -18.83, *p* < 0.001*** |
|  |  |  | 1000-1500ms | -11.96 | *t*(21) = -8.21, *p* < 0.001*** |
|  |  |  | 1500-2000ms | -11.59 | *t*(21) = -4.88, *p* < 0.001*** |
|  |  |  | 2000-2500ms | -5.50 | *t*(21) = -1.07, *p* = 0.296 |
|  |  |  | 2500-3000ms | -6.99 | *t*(21) = -1.60, *p* = 0.124 |
|  |  | incongruent | 0-500ms | -8.96 | *t*(21) = -5.59, *p* < 0.001*** |
|  |  |  | 500-1000ms | -7.24 | *t*(21) = -3.98, *p* < 0.001*** |
|  |  |  | 1000-1500ms | -4.91 | *t*(21) = -2.25, *p* = 0.035* |
|  |  |  | 1500-2000ms | -3.60 | *t*(21) = -0.93, *p* = 0.361 |
|  |  |  | 2000-2500ms | 10.94 | *t*(21) = 2.06, *p* = 0.052 |
|  |  |  | 2500-3000ms | -3.41 | *t*(21) = -0.79, *p* = 0.440 |
|  |  | scrambled | 0-500ms | -5.34 | *t*(21) = -3.69, *p* < 0.005** |
|  |  |  | 500-1000ms | -5.04 | *t*(21) = -3.75, *p* < 0.005** |
|  |  |  | 1000-1500ms | 0.78 | *t*(21) = 0.54, *p* = 0.595 |
|  |  |  | 1500-2000ms | -2.88 | *t*(21) = -1.10, *p* = 0.286 |
|  |  |  | 2000-2500ms | 2.08 | *t*(21) = 0.50, *p* = 0.625 |
|  |  |  | 2500-3000ms | 0.38 | *t*(21) = 0.10, *p* = 0.920 |
|  | 9m | congruent | 0-500ms | -27.63 | *t*(18) = -13.07, *p* < 0.001*** |
|  |  |  | 500-1000ms | -34.15 | *t*(18) = -16.91, *p* < 0.001*** |
|  |  |  | 1000-1500ms | -20.10 | *t*(17) = -10.69, *p* < 0.001*** |
|  |  |  | 1500-2000ms | -20.16 | *t*(18) = -5.53, *p* < 0.001*** |
|  |  |  | 2000-2500ms | -15.46 | *t*(17) = -3.48, *p* = 0.003** |
|  |  |  | 2500-3000ms | -15.70 | *t*(17) = -3.35, *p* = 0.004** |
|  |  | incongruent | 0-500ms | -11.82 | *t*(18) = -4.86, *p* < 0.001*** |
|  |  |  | 500-1000ms | -14.31 | *t*(18) = -8.45, *p* < 0.001*** |
|  |  |  | 1000-1500ms | -4.79 | *t*(17) = -2.34, *p* = 0.032* |
|  |  |  | 1500-2000ms | -5.48 | *t*(18) = -1.45, *p* = 0.164 |
|  |  |  | 2000-2500ms | -0.327 | *t*(17) = -0.07, *p* = 0.949 |
|  |  |  | 2500-3000ms | -5.02 | *t*(17) = -1.09, *p* = 0.289 |
|  |  | scrambled | 0-500ms | -8.56 | *t*(18) = -3.91, *p* < 0.005** |
|  |  |  | 500-1000ms | -3.84 | *t*(18) = -4.02, *p* < 0.001 |
|  |  |  | 1000-1500ms | 2.79 | *t*(17) = 1.10, *p* = 0.286 |
|  |  |  | 1500-2000ms | 3.03 | *t*(18) = 0.78, *p* = 0.443 |
|  |  |  | 2000-2500ms | 9.25 | *t*(17) = 1.28, *p* = 0.218 |
|  |  |  | 2500-3000ms | 2.50 | *t*(17) = 0.53, *p* = 0.600 |
| P3 | 6m | congruent | 0-500ms | -10.72 | *t*(21) = -1.74, *p* = 0.096 |
|  |  |  | 500-1000ms | -2.38 | *t*(21) = -0.31, *p* = 0.760 |
|  |  |  | 1000-1500ms | 7.51 | *t*(21) = 0.75, *p* = 0.459 |
|  |  |  | 1500-2000ms | -3.58 | *t*(21) = -038, *p* = 0.706 |
|  |  |  | 2000-2500ms | -9.97 | *t*(21) = -1.43, *p* = 0.167 |
|  |  |  | 2500-3000ms | -0.27 | *t*(21) = -0.04, *p* = 0.971 |
|  |  | incongruent | 0-500ms | -5.01 | *t*(21) = -0.86, *p* = 0.397 |
|  |  |  | 500-1000ms | -5.52 | *t*(21) = -1.40, *p* = 0.176 |
|  |  |  | 1000-1500ms | 0.38 | *t*(21) = 0.05, *p* = 0.957 |
|  |  |  | 1500-2000ms | -4.58 | *t*(21) = -0.91, *p* = 0.375 |
|  |  |  | 2000-2500ms | 4.82 | *t*(21) = 0.67, *p* = 0.510 |
|  |  |  | 2500-3000ms | 0.19 | *t*(21) = 0.03, *p* = 0.979 |
|  |  | scrambled | 0-500ms | -9.58 | *t*(21) = -1.78, *p* = 0.089 |
|  |  |  | 500-1000ms | -9.47 | *t*(21) = -2.21, *p* = 0.038* |
|  |  |  | 1000-1500ms | -2.75 | *t*(21) = -0.52, *p* = 0.611 |
|  |  |  | 1500-2000ms | -1.90 | *t*(21) = -0.42, *p* = 0.678 |
|  |  |  | 2000-2500ms | -2.50 | *t*(21) = -0.47, *p* = 0.641 |
|  |  |  | 2500-3000ms | 4.11 | *t*(21) = 0.70, *p* = 0.489 |
|  | 9m | congruent | 0-500ms | -20.69 | *t*(18) = -4.06, *p* < 0.001*** |
|  |  |  | 500-1000ms | -22.06 | *t*(17) = -4.50, *p* < 0.001*** |
|  |  |  | 1000-1500ms | -10.45 | *t*(18) = -1.77, *p* = 0.094 |
|  |  |  | 1500-2000ms | -12.36 | *t*(17) = 2.34, *p* = 0.032* |
|  |  |  | 2000-2500ms | -18.73 | *t*(17) = -2.98, *p* = 0.008** |
|  |  |  | 2500-3000ms | -12.16 | *t*(17) = -2.22, *p* = 0.040* |
|  |  | incongruent | 0-500ms | -16.61 | *t*(18) = -3.50, *p* = 0.003** |
|  |  |  | 500-1000ms | -17.40 | *t*(17) = -3.00, *p* = 0.008** |
|  |  |  | 1000-1500ms | 3.68 | *t*(18) = 0.62, *p* = 0.546 |
|  |  |  | 1500-2000ms | -1.51 | *t*(17) = -0.23, *p* = 0.819 |
|  |  |  | 2000-2500ms | -9.55 | *t*(17) = -1.62, *p* = 0.124 |
|  |  |  | 2500-3000ms | -0.97 | *t*(17) = -0.11, *p* = 0.917 |
|  |  | scrambled | 0-500ms | -8.93 | *t*(18) = -1.76, *p* = 0.095 |
|  |  |  | 500-1000ms | -9.21 | *t*(17) = -1.66, *p* = 0.115 |
|  |  |  | 1000-1500ms | 1.35 | *t*(18) = 0.14, *p* = 0.890 |
|  |  |  | 1500-2000ms | -11.81 | *t*(17) = -1.86, *p* = 0.080 |
|  |  |  | 2000-2500ms | -7.44 | *t*(17) = -1.25, *p* = 0.229 |
|  |  |  | 2500-3000ms | -2.39 | *t*(17) = -0.32, *p* = 0.752 |
| P4 | 6m | congruent | 0-500ms | -17.29 | *t*(21) = -5.33, *p* < 0.001*** |
|  |  |  | 500-1000ms | -17.64 | *t*(21) = -3.94, *p* < 0.001*** |
|  |  |  | 1000-1500ms | -2.97 | *t*(21) = -0.66, *p* = 0.514 |
|  |  |  | 1500-2000ms | -6.72 | *t*(21) = -1.24, *p* = 0.230 |
|  |  |  | 2000-2500ms | -6.62 | *t*(21) = -1.15, *p* = 0.263 |
|  |  |  | 2500-3000ms | -3.61 | *t*(21) = -0.80, *p* = 0.434 |
|  |  | incongruent | 0-500ms | -4.14 | *t*(21) = -0.65, *p* = 0.524 |
|  |  |  | 500-1000ms | -7.78 | *t*(21) = -1.44, *p* = 0.166 |
|  |  |  | 1000-1500ms | 8.47 | *t*(21) = 1.09, *p* = 0.287 |
|  |  |  | 1500-2000ms | 1.15 | *t*(21) = 0.15, *p* = 0.880 |
|  |  |  | 2000-2500ms | 4.06 | *t*(21) = 0.49, *p* = 0.630 |
|  |  |  | 2500-3000ms | -4.98 | *t*(21) = -1.04, *p* = 0.311 |
|  |  | scrambled | 0-500ms | -6.97 | *t*(21) = -1.42, *p* = 0.169 |
|  |  |  | 500-1000ms | -6.86 | *t*(21) = -1.47, *p* = 0.157 |
|  |  |  | 1000-1500ms | 2.84 | *t*(21) = 0.49, *p* = 0.630 |
|  |  |  | 1500-2000ms | 2.76 | *t*(21) = 0.35, *p* = 0.727 |
|  |  |  | 2000-2500ms | -2.76 | *t*(21) = -0.37, *p* = 0.718 |
|  |  |  | 2500-3000ms | -1.59 | *t*(21) = -0.24, *p* = 0.812 |
|  | 9m | congruent | 0-500ms | -19.96 | *t*(18) = -3.92, *p* = 0.001** |
|  |  |  | 500-1000ms | -19.06 | *t*(18) = -5.54, *p* < 0.001*** |
|  |  |  | 1000-1500ms | -4.71 | *t*(17) = -0.87, *p* = 0.395 |
|  |  |  | 1500-2000ms | -5.40 | *t*(17) = -0.90, *p* = 0.380 |
|  |  |  | 2000-2500ms | -6.53 | *t*(17) = -1.09, *p* = 0.291 |
|  |  |  | 2500-3000ms | -1.75 | *t*(17) = -0.35, *p* = 0.731 |
|  |  | incongruent | 0-500ms | -7.70 | *t*(18) = -0.79, *p* = 0.437 |
|  |  |  | 500-1000ms | 1.87 | *t*(18) = 0.11, *p* = 0.910 |
|  |  |  | 1000-1500ms | 3.68 | *t*(17) = 0.63, *p* = 0.540 |
|  |  |  | 1500-2000ms | -0.60 | *t*(17) = -0.11, *p* = 0.916 |
|  |  |  | 2000-2500ms | 4.86 | *t*(17) = 0.63, *p* = 0.540 |
|  |  |  | 2500-3000ms | 2.90 | *t*(17) = 0.44, *p* = 0.666 |
|  |  | scrambled | 0-500ms | -15.00 | *t*(18) = -3.56, *p* = 0.002** |
|  |  |  | 500-1000ms | -6.10 | *t*(18) = -0.94, *p* = 0.360 |
|  |  |  | 1000-1500ms | 14.11 | *t*(17) = 2.01, *p* = 0.061 |
|  |  |  | 1500-2000ms | 0.34 | *t*(17) = 0.06, *p* = 0.951 |
|  |  |  | 2000-2500ms | 10.36 | *t*(17) = 1.07, *p* = 0.298 |
|  |  |  | 2500-3000ms | 0.27 | *t*(17) = 0.04, *p* = 0.972 |
| O1 | 6m | congruent | 0-500ms | -14.19 | *t*(21) = -6.62, *p* < 0.001*** |
|  |  |  | 500-1000ms | -10.68 | *t*(21) = -5.15, *p* < 0.001*** |
|  |  |  | 1000-1500ms | -8.24 | *t*(21) = -3.20, *p* = 0.004** |
|  |  |  | 1500-2000ms | -16.55 | *t*(21) = -3.81, *p* = 0.001** |
|  |  |  | 2000-2500ms | -2.60 | *t*(21) = -0.44, *p* = 0.665 |
|  |  |  | 2500-3000ms | 3.14 | *t*(21) = 0.63, *p* = 0.539 |
|  |  | incongruent | 0-500ms | -12.64 | *t*(21) = -5.23, *p* < 0.001*** |
|  |  |  | 500-1000ms | -11.03 | *t*(21) = -4.55, *p* < 0.001*** |
|  |  |  | 1000-1500ms | -10.15 | *t*(21) = -3.98, *p* < 0.001*** |
|  |  |  | 1500-2000ms | -22.06 | *t*(21) = -4.08, *p* < 0.001*** |
|  |  |  | 2000-2500ms | -15.15 | *t*(21) = -3.82, *p* < 0.001*** |
|  |  |  | 2500-3000ms | -10.19 | *t*(21) = -1.66, *p* = 0.111 |
|  |  | scrambled | 0-500ms | -18.38 | *t*(21) = -6.67, *p* < 0.001*** |
|  |  |  | 500-1000ms | -16.63 | *t*(21) = -4.29, *p* < 0.001*** |
|  |  |  | 1000-1500ms | -10.84 | *t*(21) = -4.09, *p* < 0.001*** |
|  |  |  | 1500-2000ms | -16.25 | *t*(21) = -3.74, *p* = 0.001** |
|  |  |  | 2000-2500ms | -16.22 | *t*(21) = -3.96, *p* < 0.001*** |
|  |  |  | 2500-3000ms | -5.66 | *t*(21) = -0.84, *p* = 0.409 |
|  | 9m | congruent | 0-500ms | -16.65 | *t*(18) = -5.37, *p* < 0.001*** |
|  |  |  | 500-1000ms | -13.93 | *t*(17) = -6.56, *p* < 0.001*** |
|  |  |  | 1000-1500ms | -4.86 | *t*(18) = -1.23, *p* = 0.234 |
|  |  |  | 1500-2000ms | -22.07 | *t*(17) = -4.40, *p* < 0.001*** |
|  |  |  | 2000-2500ms | -8.37 | *t*(17) = -1.53, *p* = 0.145 |
|  |  |  | 2500-3000ms | -6.66 | *t*(18) = -1.11, *p* = 0.282 |
|  |  | incongruent | 0-500ms | -14.96 | *t*(18) = -7.18, *p* < 0.001*** |
|  |  |  | 500-1000ms | -15.72 | *t*(17) = -5.98, *p* < 0.001*** |
|  |  |  | 1000-1500ms | -4.76 | *t*(18) = -2.70, *p* = 0.015* |
|  |  |  | 1500-2000ms | -9.95 | *t*(17) = -2.02, *p* = 0.060 |
|  |  |  | 2000-2500ms | -12.37 | *t*(17) = -2.11, *p* = 0.050 |
|  |  |  | 2500-3000ms | -3.16 | *t*(18) = -0.42, *p* = 0.676 |
|  |  | scrambled | 0-500ms | -13.73 | *t*(18) = -3.27, *p* = 0.004** |
|  |  |  | 500-1000ms | -17.39 | *t*(17) = -8.89, *p* < 0.001*** |
|  |  |  | 1000-1500ms | -10.14 | *t*(18) = -3.46, *p* = 0.003** |
|  |  |  | 1500-2000ms | -15.52 | *t*(17) = -3.42, *p* = 0.003** |
|  |  |  | 2000-2500ms | -4.04 | *t*(17) = -0.58, *p* = 0.569 |
|  |  |  | 2500-3000ms | -5.15 | *t*(18) = -0.91, *p* = 0.375 |
| O2 | 6m | congruent | 0-500ms | -11.86 | *t*(21) = -3.99, *p* < 0.001*** |
|  |  |  | 500-1000ms | -11.86 | *t*(21) = -6.47, *p* < 0.001*** |
|  |  |  | 1000-1500ms | -7.66 | *t*(21) = -2.71, *p* = 0.013* |
|  |  |  | 1500-2000ms | -14.78 | *t*(21) = -3.42, *p* = 0.003** |
|  |  |  | 2000-2500ms | -7.44 | *t*(21) = -1.26, *p* = 0.221 |
|  |  |  | 2500-3000ms | -0.70 | *t*(21) = -0.08, *p* = 0.937 |
|  |  | incongruent | 0-500ms | -14.46 | *t*(21) = -6.56, *p* < 0.001*** |
|  |  |  | 500-1000ms | -10.80 | *t*(21) = -5.69, *p* < 0.001*** |
|  |  |  | 1000-1500ms | -6.54 | *t*(21) = -3.02, *p* = 0.007** |
|  |  |  | 1500-2000ms | -13.74 | *t*(21) = -2.87, *p* = 0.009** |
|  |  |  | 2000-2500ms | -12.65 | *t*(21) = -2.99, *p* = 0.007** |
|  |  |  | 2500-3000ms | -9.38 | *t*(21) = -1.72, *p* = 0.100 |
|  |  | scrambled | 0-500ms | -19.44 | *t*(21) = -10.83, *p* < 0.001*** |
|  |  |  | 500-1000ms | -13.03 | *t*(21) = -4.10, *p* < 0.001*** |
|  |  |  | 1000-1500ms | -12.90 | *t*(21) = -4.89, *p* < 0.001*** |
|  |  |  | 1500-2000ms | -12.06 | *t*(21) = -3.46, *p* = 0.002** |
|  |  |  | 2000-2500ms | -17.06 | *t*(21) = -3.93, *p* < 0.001*** |
|  |  |  | 2500-3000ms | -7.83 | *t*(21) = -1.64, *p* = 0.115 |
|  | 9m | congruent | 0-500ms | -10.26 | *t*(18) = -6.57, *p* < 0.001*** |
|  |  |  | 500-1000ms | -14.29 | *t*(18) = -7.98, *p* < 0.001*** |
|  |  |  | 1000-1500ms | -2.38 | *t*(18) = -0.80, *p* = 0.432 |
|  |  |  | 1500-2000ms | -15.96 | *t*(17) = -3.93, *p* = 0.001** |
|  |  |  | 2000-2500ms | -6.24 | *t*(17) = -1.20, *p* = 0.245 |
|  |  |  | 2500-3000ms | -1.52 | *t*(18) = -0.33, *p* = 0.747 |
|  |  | incongruent | 0-500ms | -13.53 | *t*(18) = -6.77, *p* < 0.001*** |
|  |  |  | 500-1000ms | -12.95 | *t*(18) = -6.33, *p* < 0.001*** |
|  |  |  | 1000-1500ms | -1.68 | *t*(18) = -0.90, *p* = 0.383 |
|  |  |  | 1500-2000ms | -8.35 | *t*(17) = -1.52, *p* = 0.148 |
|  |  |  | 2000-2500ms | -7.23 | *t*(17) = -1.23, *p* = 0.237 |
|  |  |  | 2500-3000ms | 4.49 | *t*(18) = 0.56, *p* = 0.581 |
|  |  | scrambled | 0-500ms | -19.42 | *t*(18) = -8.00, *p* < 0.001*** |
|  |  |  | 500-1000ms | -21.06 | *t*(18) = -11.02, *p* < 0.001*** |
|  |  |  | 1000-1500ms | -5.84 | *t*(18) = -1.63, *p* = 0.120 |
|  |  |  | 1500-2000ms | -10.48 | *t*(17) = -3.07, *p* = 0.007** |
|  |  |  | 2000-2500ms | -0.56 | *t*(17) = -0.06, *p* = 0.951 |
|  |  |  | 2500-3000ms | -4.99 | *t*(18) = -0.99, *p* = 0.337 |


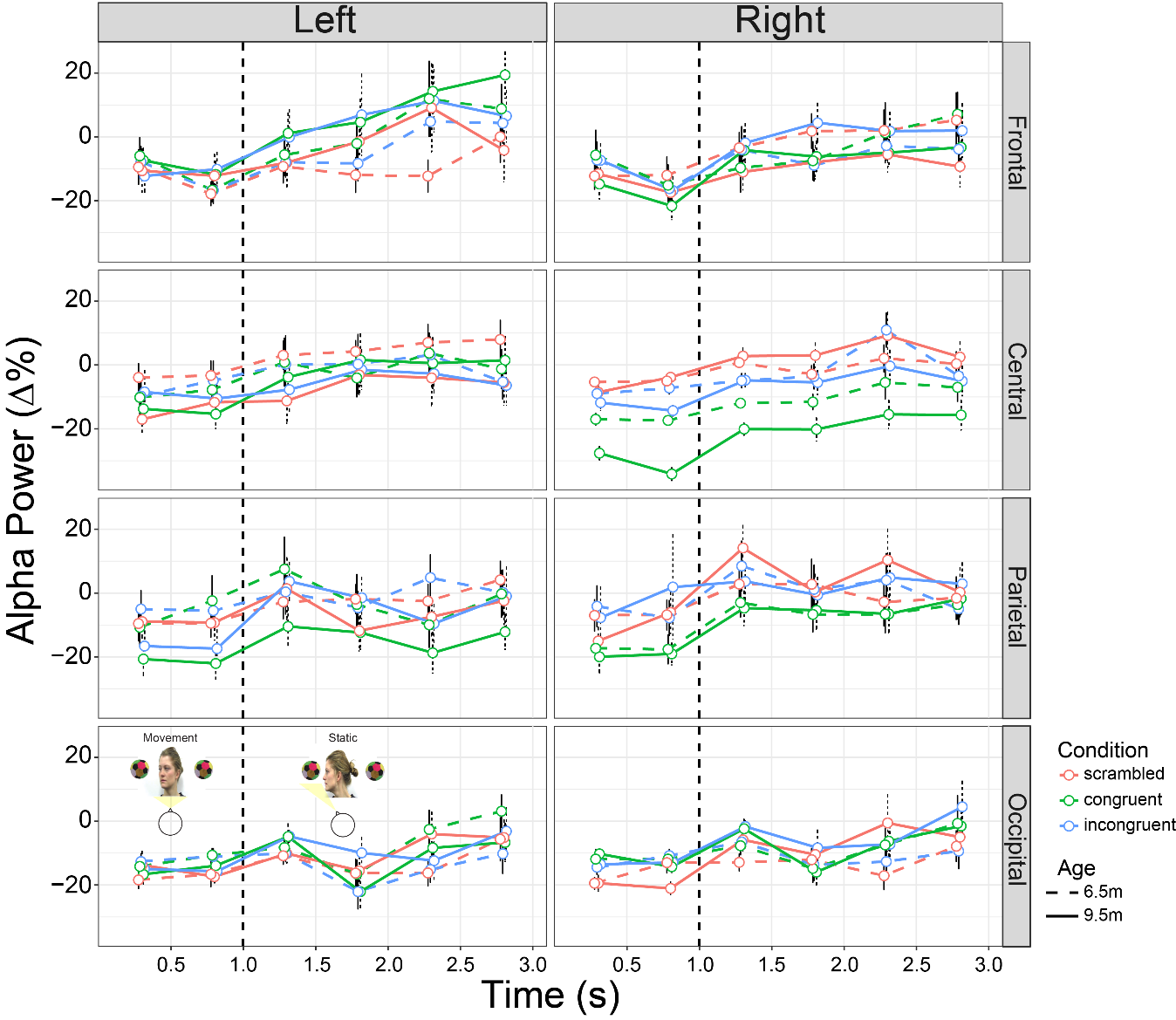


**Figure S1:** Change in alpha power from baseline in each electrode cluster at 6.5 months (dashed) and 9.5 months (solid), during observation of the three gaze following conditions. Time zero is the start of the adult actor’s head turn. The vertical dashed lines indicate the end of the observed head turn, which was followed by a static period of adult gaze towards the object. Error bars represent +/− standard error. At both ages, ERD in C4 and P4 was significantly greater in the congruent compared to incongruent and scrambled conditions, with ERD also greater at 9.5 compared to 6.5 months in C4.

***
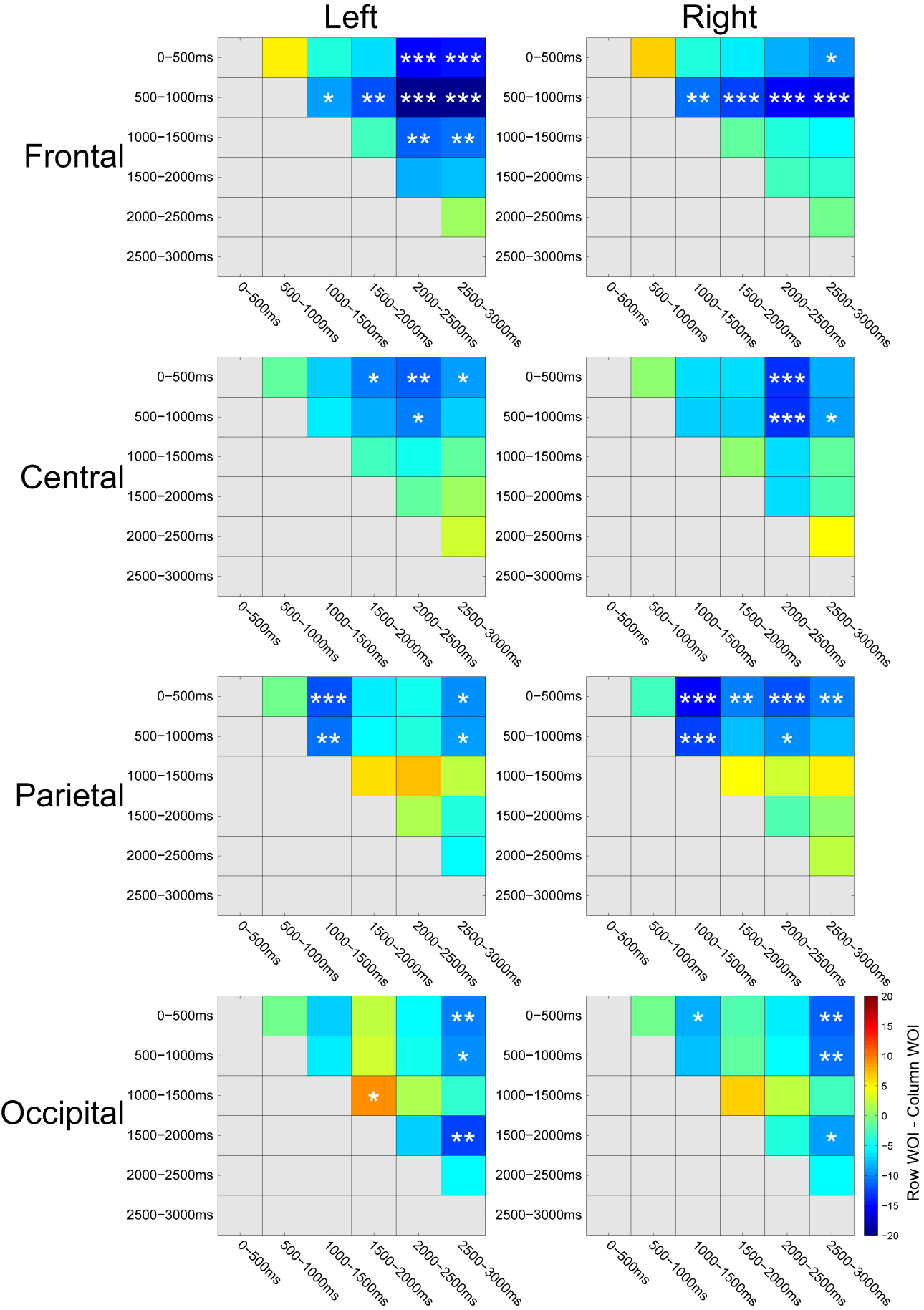
***

**Figure S2:** *Differences in baseline corrected alpha activity during observation in the various time windows of interest (WOI).* A significant interaction between electrode cluster and WOI was revealed. Planned pairwise comparisons revealed significant differences largely between early and late WOIs, with alpha power generally lower in early WOIs. Here, these differences are plotted by electrode cluster. The mean difference between WOIs is colour-coded, with blue colours representing lower alpha power in the row compared to column WOI, and vice versa for red colours. Statistically significant differences are denoted by asterisks (* *p* < 0.05, ** *p* < 0.01, *** *p* < 0.001).


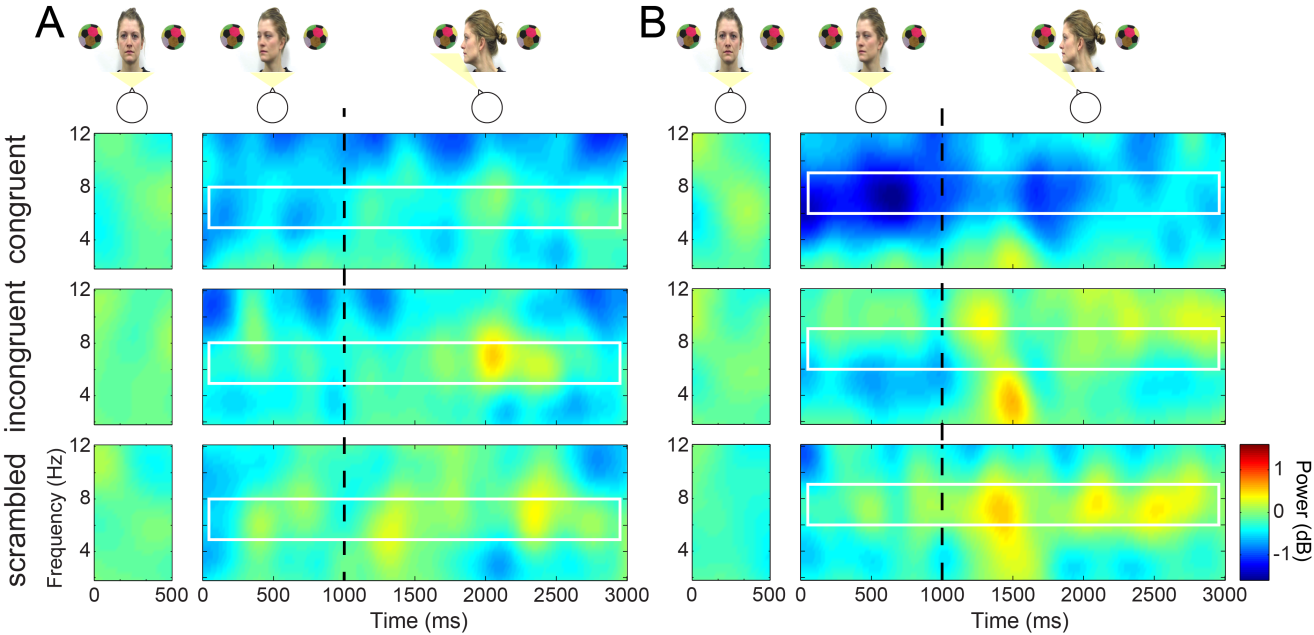


**Figure S3**: *Spectrograms of activity in the C4 cluster at 6.5m (A) and 9.5m (B) during each condition.* The baseline static period is shown in the left panel of each row and the period of the observed action is shown in the right panel. The vertical dashed lines indicate the end of the adult actor’s head turn. The white rectangles delineate the borders of the alpha frequency range that we analysed at each age.

***Infants’ own gaze behaviour during the gaze following conditions: Split by area of interest (AOI)***

Figure S4 illustrates infant gaze behaviour during EEG acquisition, in the different gaze following conditions, split by AOI: cued object (highlighted at the start of a trial), uncued object (not highlighted), and face (the adult actor).


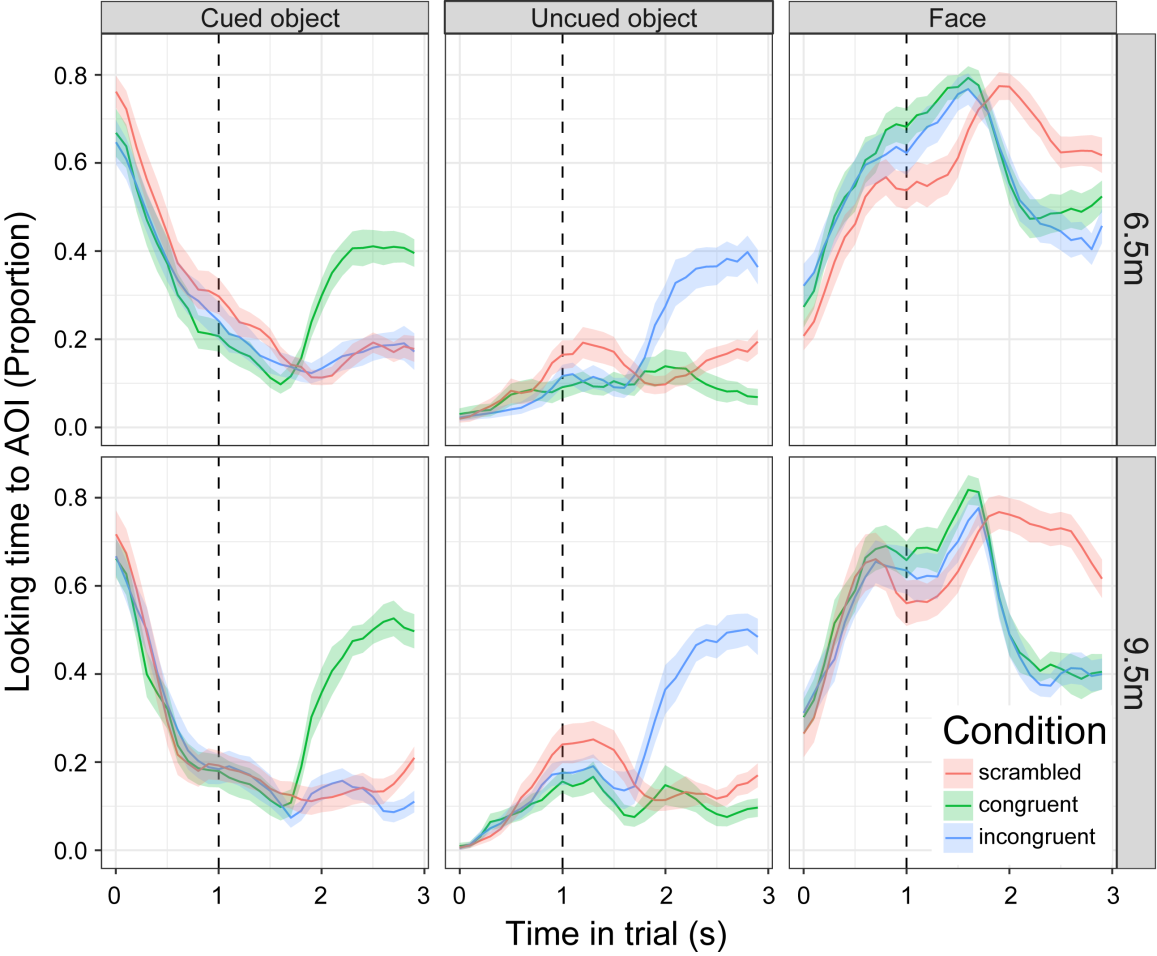


**Figure S4:** Time-course of infant gaze to the different AOIs in the three conditions, at both ages. Time zero is the start of the adult actor’s head turn. The vertical dashed lines indicate the end of the observed adult head turn, which was followed by a period of the adult looking statically to the object. Shaded regions represent +/− standard error.

***Infant gaze behaviour during EEG trials: Additional analyses***

Results from the other models run to look at the frequency or latency of gaze shifts/looking patterns during the different conditions are in keeping with the GCA results in the main manuscript. The numbers of shifts made in congruent and incongruent trials were significantly greater than in the scrambled condition (congruent – scrambled: *Z* = 4.06, *p* < 0.001; incongruent – scrambled: *Z* = 3.62, *p* < 0.001), but not from each other (*Z* = 0.43, *p* = 0.902). The only differences revealed in the looking patterns were as follows: there was a main effect of condition for the proportion of trials in which the infant’s first gaze shift after looking at the adult’s face was to the adult’s target (*χ^2^*(2) = 310.86, *p* < 0.001), with a greater proportion in the congruent and incongruent conditions compared to scrambled (congruent – scrambled: *t*(105.44) = 14.90, *p* < 0.001; incongruent – scrambled: *t*(105.44) = 15.59, *p* < 0.001); and the latency of looking from the adult’s face to the adult’s target (i.e. the cued object in the congruent condition, and the uncued object in the incongruent condition) in congruent and incongruent conditions was shorter by 9.5 months (*χ^2^*(1) = 6.40, *p* = 0.011).

***Alpha activity during infant execution of head turns***

Changes in alpha power from baseline during infants’ own head turns are shown in Figure S5. Instances of infant head turns were identified from the videos recorded during EEG acquisition.


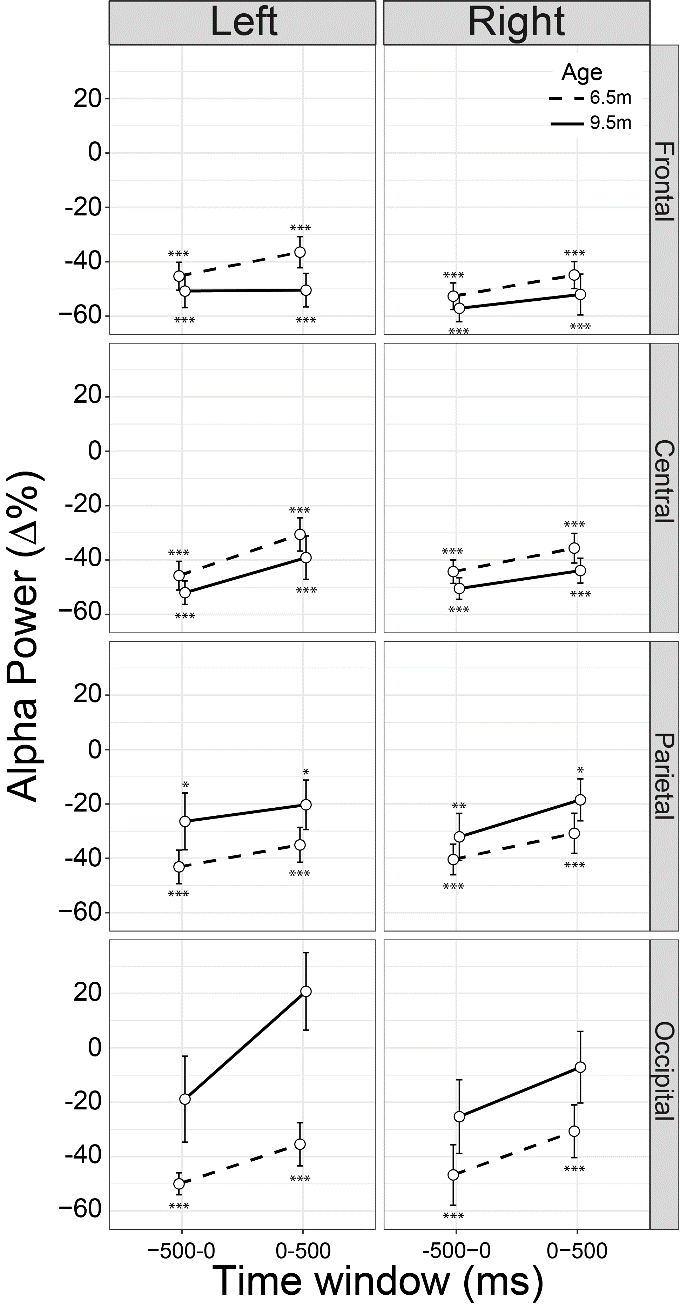


**Figure S5:** Changes in alpha power from baseline during head-turn execution (i.e. infants’ own head turns) at 6.5 and 9.5 months. Time zero corresponds to the onset of infant head-turns. At 6.5 months (dashed line), alpha power was lower in bilateral occipital and left parietal clusters compared to 9.5 months (solid line). Error bars represent +/− standard error. Significant differences from baseline are denoted by * p < 0.05; ** p < 0.01; *** p < 0.001.

A linear mixed model framework was used for statistical analysis of the execution data. Percentage change in alpha power from baseline was treated as the dependent measure, with cluster (F3/F4/C3/C4/P3/P4/O1/O2), WOI (-500-0ms/0-500ms), age (6.5/9.5 months), and their interactions as fixed effects. Subject-specific intercepts were included as random effects. Main effects of cluster (*χ^2^*(7) = 50.24, *p* < 0.001), WOI (*χ^2^*(1) = 23.47, *p* < 0.001), and age (*χ^2^*(1) = 6.02, *p* = 0.014) were revealed, qualified by an age x cluster (*χ^2^*(7) = 51.67, *p* < 0.001) interaction.

Pairwise comparisons revealed that there was significantly more alpha power in O1 (*t*(450.15) = -5.752; *p* < 0.0001), O2 (*t*(450.15) = -2.988; *p* < 0.005), and P3(*t*(450.15) = -2.107; *p* = 0.036) at 9.5 compared to 6.5 months of age. This suggests that ERD may have become more localized by 9.5 months.

***Scrambled gaze following condition split into congruent versus incongruent coherent motion: Alpha power and infant gaze***

Again, infants were required to have a minimum of five trials per condition after pre-processing of the EEG data to be included in the analysis. At 6.5 months (n = 15), there was an average of 8.73 (SD = 2.58) trials in the scrambled congruent condition, and 8.67 (SD = 3.16) in the scrambled incongruent condition. At 9.5m (n = 14 subjects), there was an average of 9.79 (SD = 2.67) trials in the scrambled congruent condition, and 9.79 (SD = 2.23) in the scrambled incongruent condition.

The linear mixed model analysis revealed a main effect of age (*χ^2^*(1) = 42.82, *p* < 0.001), electrode cluster (*χ^2^*(7) = 197.91, *p* < 0.001), and WOI (*χ^2^*(5) = 111.64, *p* < 0.001) on baseline-corrected alpha power (Figure S6). These were qualified by an age x cluster interaction (*χ^2^*(7) = 24.19, *p* < 0.005), cluster x condition interaction (*χ^2^*(7) = 35.76, *p* < 0.001), and age x cluster x condition interaction (*χ^2^*(7) = 16.95, *p* = 0.018). At 6.5m there was less alpha power in F3 during observation of congruent scrambled than incongruent scrambled (*t*(228.16) = -3.58, *p* < 0.001), but less alpha power in P4 during incongruent compared to congruent (*t*(220.56) = 2.88, *p* < 0.005). There was an increase in alpha power from 6.5m to 9.5 in the congruent scrambled condition in C4 (*t*(2884.44) = -2.25, *p* = 0.024) and F3 (*t*(2884.45) = -3.17, *p* < 0.005). There was also in increase in alpha power in O1 and O2 from 6.5m to 9.5 in congruent and incongruent scrambled conditions (O1, congruent: *t*(2884.44) = -1.98, *p* = 0.048; O2, congruent: *t*(2884.05) = -3.40, *p* < 0.001; O1, incongruent: *t*(2884.69) = -4.74, *p* < 0.001; O2, incongruent: *t*(2884.49) = -4.77, *p* < 0.001).

No difference in amount of time infants looked to the screen (*χ^2^*(1) = 2.91, *p* = 0.088) was revealed in the two conditions. A GCA analysis revealed a significant main effect of AOI (*χ^2^*(2) = 11853.21, *p* < 0.001) as well as the following interactions: between AOI and age (*χ^2^*(2) = 105.31, *p* < 0.001); AOI and the four time codes (linear, *χ^2^*(2) = 2946.36, *p* < 0.001; quadratic, *χ^2^*(2) = 1496.71, *p* < 0.001; cubic, *χ^2^*(2) = 162.44, *p* < 0.001; quartic, *χ^2^*(2) = 114.38, *p* < 0.001); AOI, age, and the four time codes (linear, *χ^2^*(2) = 42.49, *p* < 0.001; quadratic, *χ^2^*(2) = 11.20, *p* = 0.004; cubic, *χ^2^*(2) = 18.64, *p* < 0.001; quartic, *χ^2^*(2) = 18.54, *p* < 0.001); AOI, congruence and the four time codes (linear, *χ2*(2) = 20.52, *p* < 0.001; quadratic, *χ^2^*(2) = 9.24, *p* = 0.010; cubic, *χ^2^*(2) = 6.14, *p* = 0.046 ; quartic, *χ^2^*(2) = 8.85, *p* = 0.012); and AOI, congruence, age, and two time codes (linear, *χ^2^*(2) = 15.26, *p* < 0.001; quadratic, *χ^2^*(2) = 27.21, *p* < 0.001; Figures S7, S8). However, follow-up comparisons revealed no significant differences between scrambled congruent and incongruent trials (all *p* > 0.09).


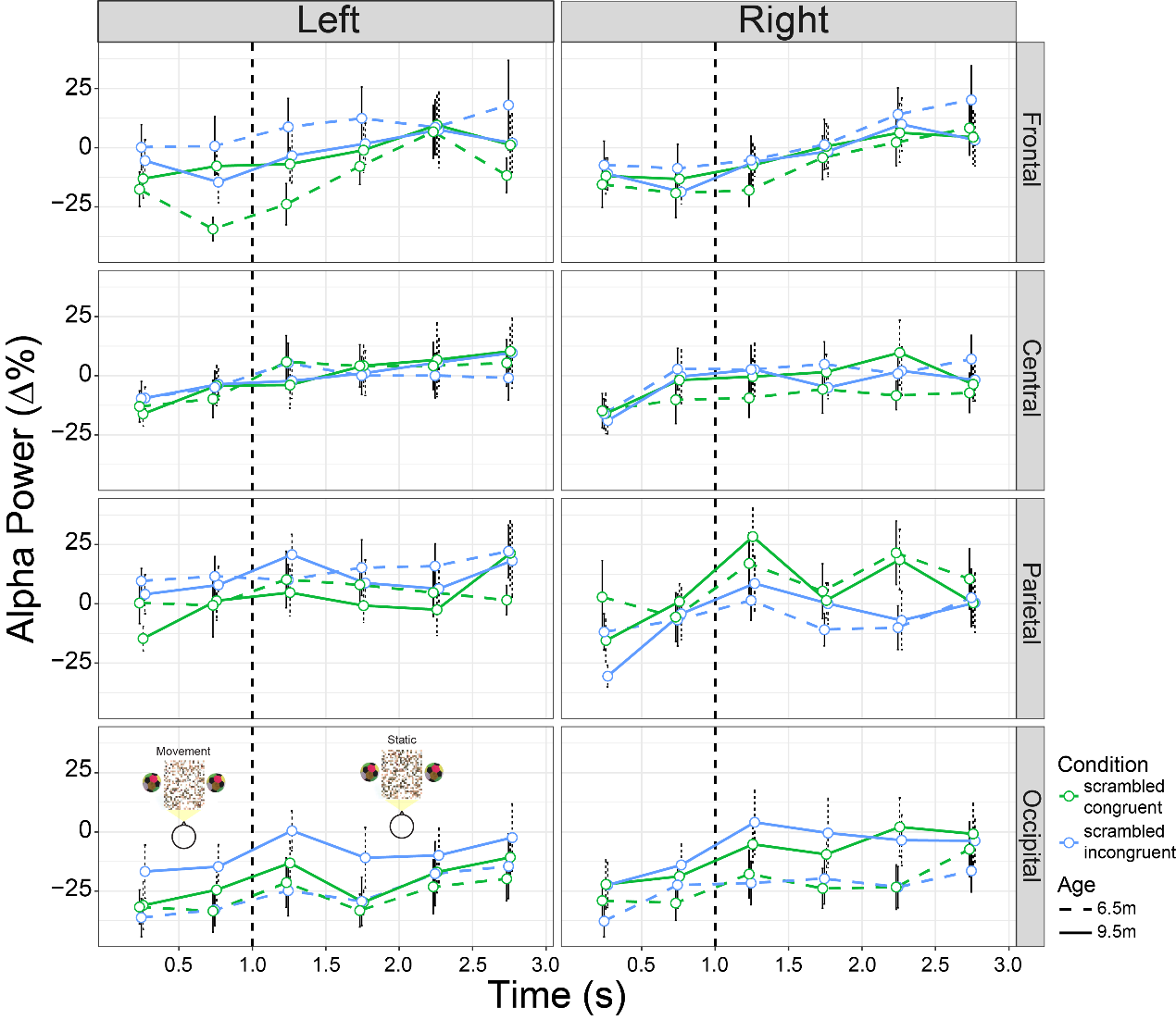


**Figure S6:** Baseline-corrected alpha power in all clusters in the congruent scrambled and incongruent scrambled trials at 6.5m (dashed) and 9.5m (solid). Time zero is the start of the adult actor’s head turn. The vertical dashed lines indicate the end of the observed head turn, which was followed by a static period of adult gaze towards the object. Error bars represent +/− standard error.


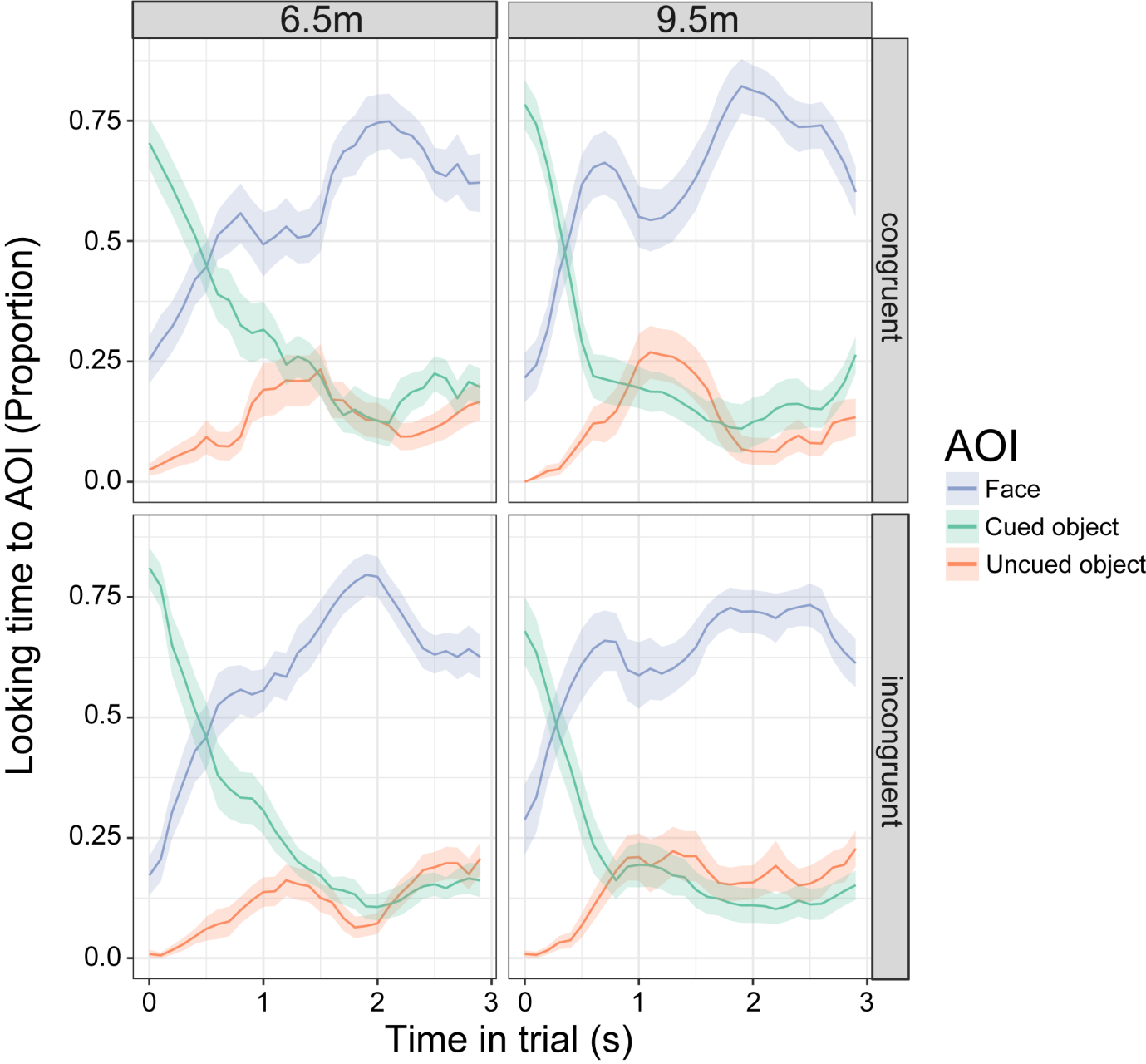


**Figure S7:** Time-course of infant gaze to the various AOIs in congruent and incongruent scrambled conditions. Shaded regions represent +/− standard error.


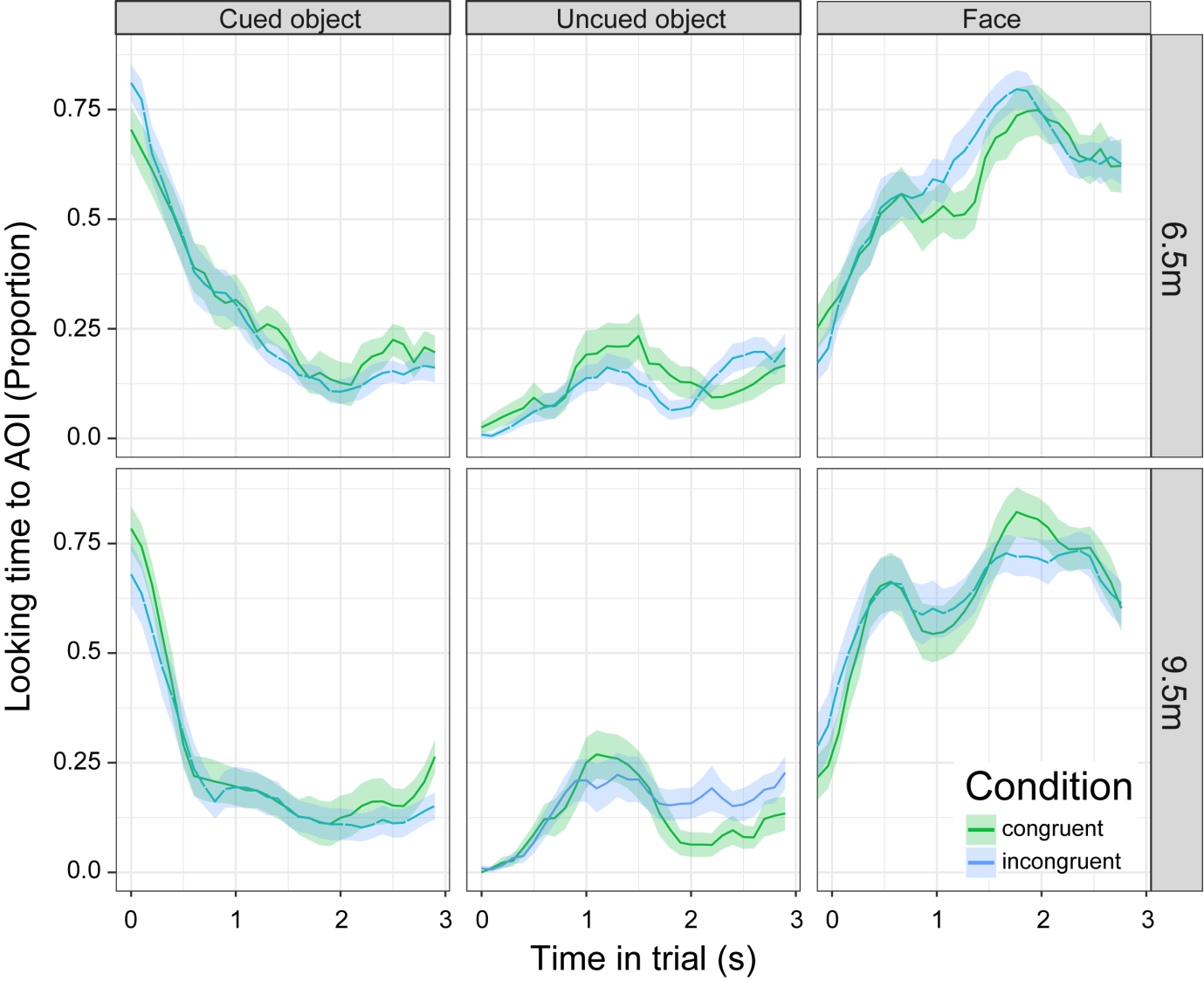


**Figure S8:** Time-course of looking to the different AOIs in congruent and incongruent scrambled conditions, split by AOI. Shaded regions represent +/− standard error.

***Analysis of fronto-medial theta power***

To determine whether infants expected adults to turn towards the cued object, and thus whether our main results might be attributable to the congruent movement being more predictable than the incongruent, we conducted an additional analysis of theta band activity (3-4Hz) in a fronto-medial cluster of electrodes (electrode numbers 9,14,15,21,22). Theta event-related synchronization (ERS) in this region has been linked to error detection in both infants and children (e.g. Berger et al., 2006; Conejero et al., 2018). Therefore, greater fronto-medial theta ERS in the incongruent condition would suggest that infants’ expectations had been violated by the adult’s response to their gaze shift.

A linear mixed model was used for statistical analysis with baseline-corrected theta power as the dependent measure and condition (congruent/incongruent/scrambled), WOI (0-500/500-1000/1000-1500ms/1500-2000ms/2000-2500ms/2500-3000ms), age (6.5/9.5 months), and their interactions as fixed effects. Subject-specific intercepts and by-subject condition slopes were included as random effects. This analysis revealed a main effect of age (χ^2^(1) = 11.16, p < 0.001), which was qualified by an interaction between age and condition (χ^2^(2) = 14.66, p < 0.001). Pairwise comparisons revealed that theta power was higher in the scrambled condition at 9.5m compared to 6.5m (t(703.16) = -4.97, p < 0.0001), and that at 9.5m theta power in the scrambled condition was higher than in the congruent (t(42.95) = 3.97, p < 0.001) and incongruent conditions (t(55.45) = 3.15, p= 0.007; Figure S9).

As no differences were found between congruent and incongruent conditions in fronto-medial theta power, it seems unlikely that the EEG results from our main analysis of alpha activity can be interpreted in terms of expectation violation. Instead, these results support the idea that congruent and incongruent conditions were equally predictable (i.e. the adults’ response was contingent on the infant’s behaviour whether or not it was congruent, and as the same adult actor always turned in a congruent or incongruent manner, infants could learn to predict the response of both actors). It is therefore more plausible that the early timing of alpha ERD in right central and parietal clusters reflects the prediction of the adult’s matching response.

Greater theta ERS in the scrambled condition could reflect greater sustained attention during this condition due to the novelty of the stimulus (e.g. Orekhova et al., 1999). This interpretation is supported by the longer looking time to the scrambled versus unscrambled faces revealed in the analysis of infant gaze behaviour during EEG trials.


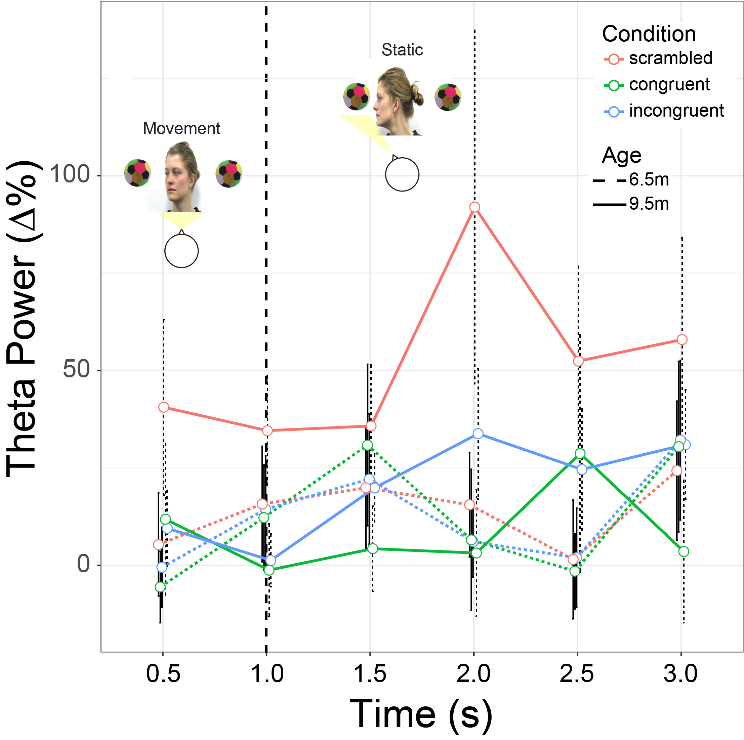


**Figure S9**: *Baseline-corrected* *theta power in the fronto-medial electrode cluster over time at 6.5 months (dashed lines) and 9.5 months (solid lines) during the scrambled (red), congruent (green), and incongruent (blue) conditions*. Time zero is the start of the adult actor’s head turn. The vertical dashed line indicates the end of the observed head turn, which was followed by a static period of adult gaze towards the object. Error bars represent +/− standard error.

**REFERENCES**

Berger, A., Tzur, G., Posner, M.I., 2006. Infant brains detect arithmetic errors. Proc. Natl. Acad. Sci. U. S. A. 103, 12649–53. https://doi.org/10.1073/pnas.0605350103

Bigdely-Shamlo, N., Mullen, T., Kothe, C., Su, K.-M., Robbins, K.A., 2015. The PREP pipeline: standardized preprocessing for large-scale EEG analysis. Front. Neuroinform. 9, 16. https://doi.org/10.3389/fninf.2015.00016

Cannon, E., Simpson, E.A., Fox, N.A., Vanderwert, R.E., Woodward, A.L., Ferrari, P., 2016. Relations between infants’ emerging reach-grasp competence and event-related desynchronization in EEG. Dev. Sci. 19, 50–62. https://doi.org/10.1111/desc.12295

Conejero, Á., Guerra, S., Abundis-Gutiérrez, A., Rueda, M.R., 2018. Frontal theta activation associated with error detection in toddlers: influence of familial socioeconomic status. Dev. Sci. 21, e12494. https://doi.org/10.1111/desc.12494

Delorme, A., Makeig, S., 2004. EEGLAB: an open source toolbox for analysis of single-trial EEG dynamics including independent component analysis. J. Neurosci. Methods 134, 9–21. https://doi.org/10.1016/j.jneumeth.2003.10.009

Farneback, G., 2000. Fast and accurate motion estimation using orientation tensors and parametric motion models, in: Proceedings 15th International Conference on Pattern Recognition. ICPR-2000. IEEE Comput. Soc, pp. 135–139. https://doi.org/10.1109/ICPR.2000.905291

Marshall, P.J., Saby, J.N., Meltzoff, A., 2013. Imitation and the Developing Social Brain: Infants’ Somatotopic EEG Patterns for Acts of Self and Other. Int. J. Psychol. Res. 6, 22–29.

Mognon, A., Jovicich, J., Bruzzone, L., Buiatti, M., 2011. ADJUST: An automatic EEG artifact detector based on the joint use of spatial and temporal features. Psychophysiology 48, 229–40. https://doi.org/10.1111/j.1469-8986.2010.01061.x

Orekhova, E. V, Stroganova, T.A., Posikera, I.N., 1999. Theta synchronization during sustained anticipatory attention in infants over the second half of the first year of life. Int. J. Psychophysiol. 32, 151–72.

Rayson, H., Bonaiuto, J.J., Ferrari, P.F., Murray, L., 2017. Early maternal mirroring predicts infant motor system activation during facial expression observation. Sci. Rep. 7. https://doi.org/10.1038/s41598-017-12097-w

Southgate, V., Johnson, M.H., Osborne, T., Csibra, G., 2009. Predictive motor activation during action observation in human infants. Biol. Lett. 5, 769–72. https://doi.org/10.1098/rsbl.2009.0474
